# Multi-omics analysis reveals ACOT1 as the key target of piperine in *Piper Longum*-mediated gastric cancer treatment

**DOI:** 10.1186/s13020-025-01186-y

**Published:** 2025-08-25

**Authors:** Boyu Pan, Ling Liu, Xiaofeng Wang, Runfang Wang, Zeyang Liu, Xiaoyan Li, Bao Jin, Jie Zhang, Rui Li, Liren Liu, Chunnuan Wu

**Affiliations:** 1 Department of Pharmacy, Key Laboratory of Cancer Prevention and Therapy, State Key Laboratory of Neurology and Oncology Drug Development, Tianjin’s Clinical Research Center for Cancer, Tianjin Medical University Cancer Institute and Hospital, National Clinical Research Center for Cancer, Tianjin, 300060 China; 2 Department of Molecular Pharmacology, Key Laboratory of Cancer Prevention and Therapy, Tianjin Medical University Cancer Institute and Hospital, National Clinical Research Center for Cancer, Tianjin’s Clinical Research Center for Cancer, Tianjin, 300060 China; 3 Department of Physics, School of Basic Medicine, Shanxi Medical University, Taiyuan, 030001 Shanxi China; 4 Inner Mongolia Autonomous Region, Xilingol Mongolian Hospital, Xilinhot, 026300 China; 5 Department of Biochemistry and Molecular Biology, Tianjin Medical University Cancer Institute and Hospital, Tianjin’s Clinical Research Center for Cancer, Tianjin, 300060 China

**Keywords:** *Piper Longum*, Multi-omics, Gastric cancer (GC), Piperine, ACOT1

## Abstract

**Background:**

*Piper longum* demonstrates significant therapeutic potential against gastric cancer (GC), but its underlying mechanisms remain incompletely understood. This study aimed to establish a comprehensive multi-omics framework to elucidate *Piper longum*'s anti-cancer mechanisms.

**Methods:**

We integrated in vivo experiments, metabolomics, gut microbiota analysis, mass spectrometry, and network pharmacology to investigate *Piper longum*’s effects. In vivo studies assessed its dose-dependent inhibition of GC growth compared to standard chemotherapy (L-OHP + 5-FU). Metabolomics identified altered lipid metabolism pathways, while gut microbiota analysis evaluated its impact on microbial composition. Piperine was identified as the key active compound, and ACOT1 was pinpointed as a critical molecular target through integrated analysis.

**Results:**

*Piper longum* significantly suppressed gastric cancer (GC) growth in a dose-dependent manner, with high-dose treatment demonstrating superior efficacy compared to conventional chemotherapy (L-OHP + 5-FU). Metabolomic analysis revealed that its anti-cancer mechanism primarily involves the reprogramming of lipid metabolism pathways in GC cells, while gut microbiota assessment confirmed that it modulates intestinal flora composition without compromising microbial diversity, supporting its favorable safety profile. Mass spectrometry identified piperine as the key bioactive compound, and integrated metabolomics and network pharmacology further pinpointed ACOT1 as a critical molecular target, which interacts with piperine that confirmed by CETSA. Notably, high ACOT1 expression was associated with poor prognosis in GC patients, underscoring its therapeutic relevance.

**Conclusions:**

This study elucidates *Piper longum*'s "component-target-pathway" mechanism in GC treatment, highlighting piperine-ACOT1-de novo lipogenesis regulatory pathway as a critical axis. Additionally, it establishes a robust multi-omics framework for evaluating traditional medicine efficacy, providing a theoretical foundation for *Piper longum*’s clinical application in GC therapy.

**Supplementary Information:**

The online version contains supplementary material available at 10.1186/s13020-025-01186-y.

## Background

Gastric cancer (GC) represents one of the most prevalent and lethal malignancies worldwide, ranking as the fifth most common cancer and the third leading cause of cancer-related mortality globally [1, 2]. Despite advances in diagnostic techniques and therapeutic strategies, the 5-year survival rate for patients with advanced gastric cancer remains dismal, rarely exceeding 30% [3]. Current standard treatments, including surgical resection, chemotherapy, and targeted therapy, are often limited by low response rates, severe adverse effects, and the development of drug resistance [2, 4]. These therapeutic challenges underscore the urgent need for novel, effective, and less toxic treatment options for gastric cancer [5].

Traditional medicinal plants have long served as valuable resources for anticancer drug discovery, with approximately 60% of currently approved anticancer drugs derived from natural sources [6–11]. Among these traditional remedies, *Piper Longum* has garnered significant attention due to its documented use in various traditional medicine systems for treating gastrointestinal disorders and cancer [12, 13]. However, the precise molecular mechanisms underlying its potential therapeutic effects in GC remain largely unexplored, limiting its development as a standardized treatment option.

Recent advances in cancer biology have highlighted the importance of metabolic reprogramming and host-microbiome interactions in GC progression [14–18]. Cancer cells exhibit altered metabolism characterized by enhanced lipid synthesis to support rapid proliferation [19], while the gut microbiota plays crucial roles in carcinogenesis and treatment response through immune modulation and metabolite production [20]. However, the interplay between these biological systems in the context of GC treatment remains poorly understood.

Piperine, the major alkaloid component of *Piper Longum*, has been reported to exhibit various pharmacological activities, including anticancer effects in several cancer models [21, 22]. However, the specific molecular targets and signaling pathways mediating piperine's effects in GC have not been systematically investigated, and its relationship with metabolic alterations and microbiota modulation remains unclear.

In this study, we employed a comprehensive multi-omics approach integrating in vivo xenograft models, untargeted metabolomics, 16S rRNA sequencing of gut microbiota, and network pharmacology analysis to investigate *Piper Longum*'s therapeutic potential in GC. Our objectives were to evaluate its anti-cancer efficacy, characterize treatment-induced alterations in metabolic pathways and gut microbiota composition, and identify key molecular targets mediating its therapeutic effects.

Our integrated analysis revealed that Piper Longum exerts potent anti-gastric cancer effects by reprogramming lipid metabolism pathways and modulating gut microbiota composition, with Acyl-CoA Thioesterase 1 (ACOT1) serving as a critical molecular target of piperine. This study not only elucidates the molecular basis for the traditional use of *Piper Longum* in cancer treatment but also identifies promising therapeutic targets for GC, potentially addressing the critical need for more effective and less toxic treatment options.

## Methods

### Cell culture

The human gastric cancer cell lines HGC-27 and AGS were procured from the China Infrastructure of Cell Line Resources. Both cell lines were cultured in DMEM (Gibco, USA; Cat#C11995500CP) supplemented with 10% FBS (v/v) (Oricell, NZL, Cat# ATPS-10001) and 100 units/mL penicillin/streptomycin (Biosharp, China; Cat# BL302A).

### Mouse xenograft model

Four-week-old male BALB/c-nude mice were obtained from Beijing Vital River Laboratory Animal Technology Co., Ltd. All animal experimental procedures were conducted in accordance with the guidelines of the Ethics Committee of Tianjin Medical University Cancer Hospital. A total of 24 mice were randomly divided into 4 groups, with 6 mice in each group. MFC cells (1.5 × 10^6^) were subcutaneously injected into the left inguinal region. After 16 days post-injection, when tumors were visible, mice were orally administered either 200 µL of saline (control group) or *Piper Longum* extract (high dose: 187.5 mg/kg body weight; low dose: 62.5 mg/kg body weight) twice daily. Concurrently, the positive control group received intraperitoneal injections of oxaliplatin (L-OHP, 4 mg/kg body weight) and 5-fluorouracil (5-FU, 20 mg/kg body weight) every three days. Tumor volume was measured daily using the formula: long diameter × (short diameter) ^2^/2, and body weight was also recorded daily. On day 12, all mice were euthanized, and tumor specimens were photographed and weighed. No accidental deaths occurred during the experiment. All animal procedures were approved by the Ethical Committee of the Tianjin Medical University Cancer Institute and Hospital (Approval No.: 2024016).

### Western blot

Mouse tumor tissues (0.1 g) were ground into powder using liquid nitrogen and lysed with RAPI buffer for protein extraction. Subsequent steps were as per previous studies. Western blot experiments were performed using specific antibodies as detailed in Table S1.

### RT-qPCR

Mouse tumor tissues (0.1 g) were ground into powder using liquid nitrogen and lysed with Trizol for RNA extraction. Subsequent steps were as per previous studies. Details of the primer sequences employed in the amplification are listed in Table S2.

### Cellular thermal shift assay (CETSA)

Cells (3 × 10^7^) were lysed with 1.5 mL NP-40 buffer containing protease inhibitors. The lysate was divided into two aliquots, treated with DMSO or piperine (60 μM), and rotated at 4 °C for 5 h. Each lysate was then divided into six parts, subjected to a temperature gradient from 35–60 °C for 3 min, followed by 3 min on ice. Finally, the samples were analyzed by western blot.

### LC–MS analysis of Piper Longum blood components

The blood components of *Piper longum* were analyzed using LC–MS following established protocols as outlined in prior research [15]. Identification and annotation of compounds were achieved by cross-referencing precise mass data, isotopic patterns, and MS/MS fragmentation spectra with various reference databases. These comprised a proprietary Traditional Chinese Medicine (TCM) standards database (Shanghai Applied Protein Technology Co., Ltd., Shanghai, China) and publicly accessible repositories such as GNPS [23], ReSpect [24], and Massbank [25].

### Metabolic pathway analysis and target screening

Untargeted metabolomic profiling of polar metabolites was performed using a quadrupole time-of-flight mass spectrometer (Sciex TripleTOF 6600) coupled with hydrophilic interaction chromatography and electrospray ionization at Shanghai Applied Protein Technology Co., Ltd. The experimental workflow adhered to established protocols as described previously [15]. Raw MS data (wiff.scan files) were converted to MzXML format using ProteoWizard MS Convert and subsequently processed with XCMS software, following standardized methodologies [15].

To assess the biological relevance of differentially expressed metabolites, the MetaboAnalyst platform was utilized for pathway enrichment analysis, which revealed potential biological functions and metabolic pathways linked to these metabolites. A metabolite-target interaction network was constructed using MetScape, integrating metabolomic data with associated protein targets. Additionally, a protein–protein interaction (PPI) network was generated, and the MCODE plugin in Cytoscape was employed to identify highly interconnected clusters through topological clustering analysis. This integrated strategy facilitated the identification of critical metabolic pathways and potential therapeutic targets.

### Gut microbiota analysis

Fecal samples were collected from tumor-bearing mice in both the control group and the BB-treated group for DNA extraction and library preparation. Following DNA extraction, representative sequences of Amplicon Sequence Variants (ASVs) were selected using the QIIME 2 software package. These sequences were then annotated by aligning them against reference databases: the Silva database (version 138) for 16S rRNA and 18S rRNA gene sequences. Taxonomic annotation was performed using the classify-sklearn tool with default parameters. Alpha and beta diversity indices were calculated to assess microbial community diversity. Alpha diversity was used to evaluate the microbial diversity within individual samples (within-community diversity).

For functional prediction of the microbial communities, PICRUSt2 (Phylogenetic Investigation of Communities by Reconstruction of Unobserved States) was employed. This tool leverages marker gene sequences (16S/18S) to predict functional abundance profiles. By utilizing the phylogenetic tree of Operational Taxonomic Units (OTUs) from the Greengenes database and associated gene information, PICRUSt2 infers the gene functional profiles of common ancestors and predicts the functional potential of unobserved species within the archaeal and bacterial domains. The resulting gene functional prediction profiles were then mapped to the sequenced microbial composition to predict metabolic functions of the gut microbiota.

### Integrated analysis of gut microbiota and metabolomics

To explore the relationship between gut microbiota and metabolomic profiles, a combined analysis was performed. First, the relative abundance of 193 bacterial genera showing significant differences (identified through 16S rDNA amplicon sequencing with LEfSe LDA > 2 and *p*-value < 0.05) and the expression levels of 84 significantly differential metabolites (identified via metabolomic analysis with VIP > 1 and *t*-test *p*-value < 0.05) were compiled from all experimental samples. Subsequently, Spearman correlation analysis was employed to calculate the correlation coefficients between the significantly differential bacterial genera and the significantly differential metabolites.

### Expression and prognostic analysis of core therapeutic targets in GC

The prognostic relevance of ACOT1 expression was investigated using the TCGA dataset, which included 370 GC patients. Patients were categorized into high- and low-expression groups based on the median ACOT1 expression level. Overall survival (OS) was evaluated using Kaplan–Meier analysis, with statistical significance assessed via the log-rank test. The hazard ratio (HR) and its 95% confidence interval (CI) were derived from a Cox proportional hazards regression model. An HR > 1 indicated that higher ACOT1 expression correlated with worse prognosis, whereas an HR < 1 suggested a protective role. The median survival time was defined as the time point at which the survival probability reached 50%. To illustrate the instantaneous risk of death over time, the cumulative hazard function was plotted, with time (years) on the x-axis and cumulative hazard on the y-axis.

### Molecular docking analysis of potential small molecules

The chemical structures of the top five small-molecule compounds exhibiting the lowest connectivity scores were obtained from the PubChem database (http://pubchem.ncbi.nlm.nih.gov/). Crystal structures of target proteins linked to hub genes were retrieved from the Protein Data Bank (PDB, http://www.wwpdb.org/). Pro-tein and ligand structures were prepared using Molecular Operating Environment (MOE) 2019, including solvent removal, protonation state optimization, hydrogen ad-dition, and charge assignment. Molecular docking analysis was then performed to cal-culate binding free energy and investigate ligand-target interactions. Results were vis-ualized using PyMOL (v 2.6.0) and Discovery Studio 2019.

### Molecular dynamics simulations

MD simulations were conducted using Gromacs2022.3, with the GAFF force field integrated via AmberTools22 for small molecule preprocessing. Simulations were conducted at a constant temperature of 300 K and pressure of 1 bar, utilizing the Amber99sb-ildn force field and the Tip3p water model. The addition of Na⁺ ions achieved system neutrality. The system underwent energy minimization using the steepest descent method, followed by equilibration under NVT and NPT ensembles for 100,000 steps each, with a coupling constant of 0.1 ps and a total duration of 100 ps. The MD simulation was performed for 5,000,000 steps with a step length of 2 fs, totaling 100 ns. Results were visualized using GraphPad Prism (v9.5) and included analyses of root mean square deviation (RMSD), root mean square fluctuation (RMSF), radius of gyra-tion (Rg), solvent-accessible surface area (SASA), and hydrogen bonds. Gibbs free en-ergy was computed using a bash script that utilized RMSD and Rg values, and the re-sulting 3D and 2D energy landscapes were created with Origin 2021.

### DFT simulation/computational details

The quantum chemical calculation was performed by DFT method using the Gaussian 09 package. The optimized geometry and electronic properties of the *piperine* molecule were calculated using the 6-31G (d,p) basis set. DFT approximated electron–electron interactions as a background functional of electronic density, and the functional is generally split into 2 parts, the Hartree potential and the exchange–correlation potential. The well-known Becke 3-Lee–Yang–Parr (B3LYP) [26] was chosen as the exchange–correlation potential. All structures were calculated at all possible spin states in this work. Molecular electrostatic potential (ESP) and the energy level of HOMO and LUMO [27], Fukui Function (f^−^) [28], Localized orbital locator (LOL) [29] and electron density of states (DOS) were calculated by Multiwfn, and VMD version 1.9.3 was utilized to visualize the shape of ESP, HOMO and LUMO.

### ACOT1 knockdown functional experiments

We performed ACOT1 knockdown in our system using the following targeting sequences: AAGGTCCAGGAGTTGGGCTGC, AACCTGGGCCCTTTCCTGGCA. These sequences were cloned into the pLKO.1 vector, and lentiviral particles were packaged using the helper plasmids PAX8 and pVSVG. The packaged viruses were then used to infect HGC-27 and AGS cell lines.

For the growth curve assay, cells were seeded at a density of 10,000 cells/mL, while the colony formation assay used 1,000 cells/well. Cells were cultured continuously for one week, followed by staining, observation, and statistical analysis [15].

### Statistical analysis

All experimental data were analyzed using GraphPad Prism software (version 6.0). Results are presented as mean ± standard error of the mean (SEM). Statistical comparisons between groups were performed using Student's *t*-test for paired data. Differences were considered statistically significant at *p* < 0.05.

## Results

### *Piper Longum significantly inhibits GC cell growth *in vivo

**Fig. 1:**
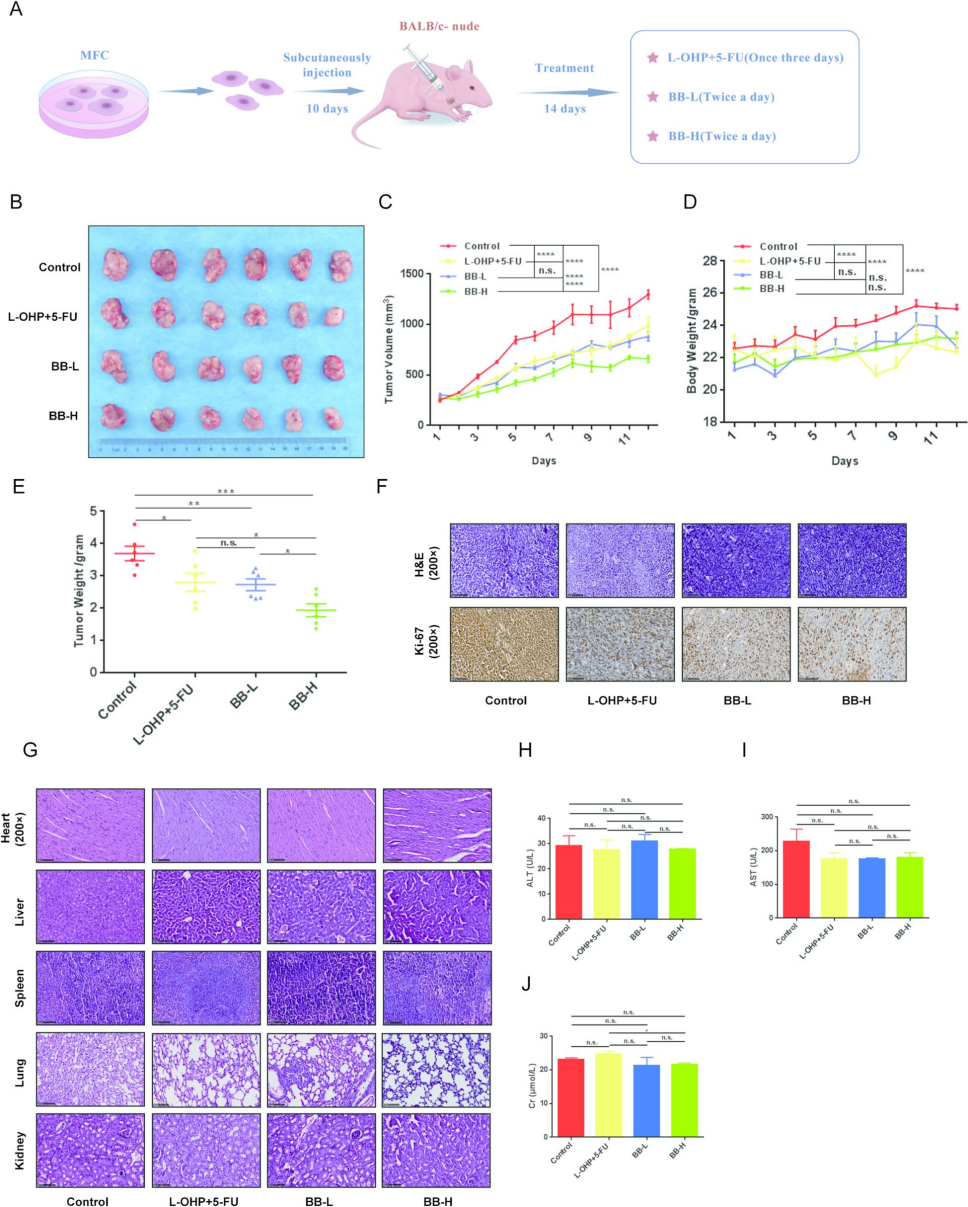
*Piper Longum* Exhibits Potent Anti-tumor Effects in a GC Xenograft Model. **A**. Schematic illustration of the experimental strategy for the xenograft model and the treatment regimen administered to tumor-bearing mice. BB-L represents the low-dose group of *Piper Longum*. BB-H represents the high-dose group of *Piper Longum*. **B**. Photographs showing tumor sizes in the treatment and control groups after 12 days of administration. **A**. Schematic illustration of the experimental strategy for the xenograft model and the treatment regimen administered to tumor-bearing mice. BB-L represents the low-dose group of *Piper Longum*. BB-H represents the high-dose group of *Piper Longum*. **B**. Photographs showing tumor sizes in the treatment and control groups after 12 days of administration. **C**. Tumor growth curves depicting changes in tumor volume over the 12-day treatment period. **D**. Body weight changes in tumor-bearing mice before and after treatment, assessing potential systemic toxicity of *Piper longum*. **E**. Tumor weight measurements at the end of the experiment. **F**. Immunohistochemical analysis: Top panel: H&E staining showing tumor tissue morphology. Bottom panel: Ki-67 staining to evaluate tumor proliferation. **G**. Toxicity evaluation in tumor-bearing mice: H&E staining of heart, liver, spleen, lung, and kidney tissues. **H**-**J**. Biochemical indicators for drug safety evaluation: **H**: Alanine aminotransferase (ALT), **I**: Aspartate aminotransferase (AST), **J**: Myocardial enzyme levels

To systematically evaluate the in vivo pharmacodynamic effects of *Piper Longum*, we established a GC xenograft model by subcutaneously inoculating mouse-derived GC MFC cells (1.5 × 10^6^ cells/mouse) into the left upper limb of BALB/c-nude mice. Following tumor formation approximately 16 days post-inoculation (diameter ~ 1 cm), mice were allocated to four treatment groups: blank control (saline), positive control (clinical first-line chemotherapy combination: L-OHP + 5-FU), low-dose *Piper Longum* (BB-L), and high-dose *Piper Longum* (BB-H) (Fig. 1A). The *Piper Longum* extracts were administered via oral gavage twice daily. Upon completion of the 28-day treatment regimen, all tumor tissues were harvested for analysis. Macroscopic examination revealed that *Piper Longum* treatment significantly inhibited GC tumor growth compared to the control group (Fig. 1B). Quantitative assessment demonstrated that tumor volumes in the high-dose treatment groups were reduced by approximately 50% (*p* < 0.05) (Fig. 1C). Notably, the high-dose *Piper Longum* group also exhibited greater tumor suppression than the positive control chemotherapy group, indicating a dose-dependent therapeutic effect (Fig. 1C). Statistical analysis of tumor weights confirmed significant differences between treatment groups (*p* < 0.01) (Fig. 1D), with the high-dose *Piper Longum* treatment demonstrating marginally superior efficacy compared to standard chemotherapy (Fig. 1D). Furthermore, body weight monitoring throughout the experiment revealed no significant weight change in treated animals, suggesting comparable systemic toxicity of *Piper Longum*-treated animals to chemotherapy-treated counterparts (Fig. 1E).

Histopathological analysis using hematoxylin and eosin (H&E) staining complemented by Ki-67 immunohistochemistry revealed progressively decreased Ki-67 expression in the *Piper Longum*-treated tumors, with staining intensity inversely correlating with *Piper Longum* concentration (Fig. 1F). This further validates the antiproliferative effect of *Piper Longum* on GC cells. To assess potential off-target toxicity, we conducted comprehensive histological examination of vital organs (heart, liver, spleen, lungs, and kidneys). Upon careful evaluation of the H&E staining results, we observed minor morphological variations in lung and liver tissue architecture between control and treatment groups. However, these changes do not represent pathological alterations or toxicity but fall within normal physiological variation ranges. The tissue architecture remained fundamentally intact, with no evidence of cellular damage, inflammatory infiltration, or structural disruption (Fig. 1G). Overall, the histological examination revealed no significant pathological alterations or toxic side effects in *Piper Longum*-treated groups, confirming the favorable safety profile of the treatment. (Fig. 1G). Additionally, liver function parameters in tumor-bearing mice remained unaffected within normal ranges across all treatment groups, further confirming the favorable safety profile of *Piper Longum* treatment (Fig. 1H-J).

### Piper Longum reprograms metabolic pathways in GC Mice

**Fig. 2:**
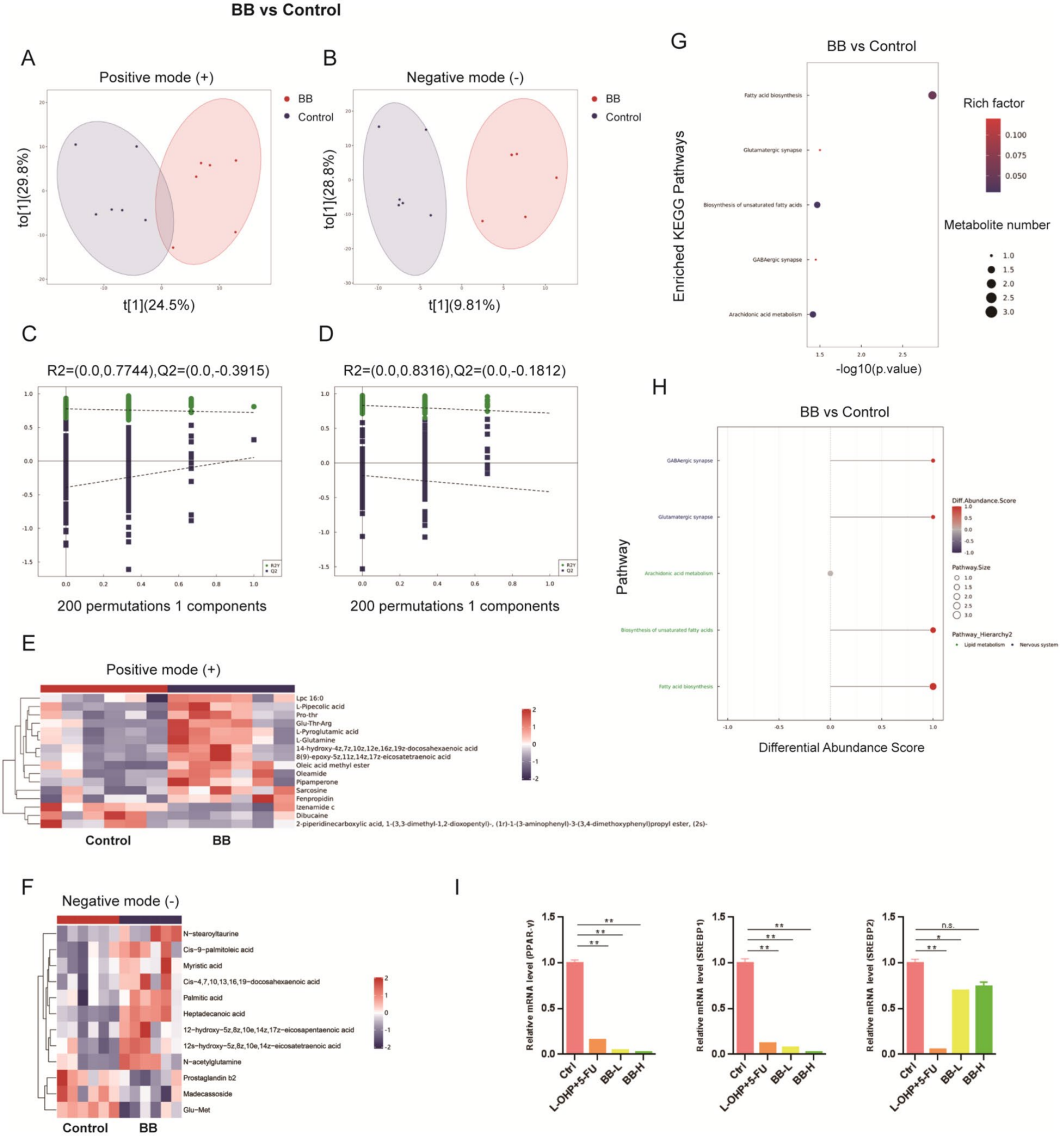
*Piper Longum* Remodels Metabolic Programming at the In Vivo Level. **A**, **B**. Orthogonal partial least-squares discriminant analysis (OPLS-DA) score plots in positive ion mode (**A**) and negative ion mode (**B**). **C**, **D**. Permutation tests for OPLS-DA score plots in positive ion mode (**C**) and negative ion mode (**D**). **E**, **F**. Heatmap visualization of differential metabolites in positive ion mode (**E**) and negative ion mode (**F**). **G**. KEGG enrichment analysis of differential metabolites. **H**. KEGG pathway-level enrichment analysis of differential metabolites. **I**. RT-qPCR analysis of lipid metabolism-related genes in tumor tissues (*p < 0.05, **p < 0.01)

Having established the inhibitory effects of *Piper Longum* on gastric cancer progression in vivo, we sought to elucidate the underlying molecular mechanisms through comprehensive metabolomic analysis. We collected blood from the orbital sinus of tumor-bearing mice immediately prior to euthanasia. Plasma samples from both the control and high-dose *Piper Longum* groups (n = 6 per group) were subjected to untargeted metabolomic profiling (Fig. 1A). Orthogonal partial least squares-discriminant analysis (OPLS-DA) was performed for quality control and visualization of metabolic profiles. The resultant score plots revealed distinct clustering and clear separation between control and *Piper Longum*-treated groups in both positive and negative ion modes, demonstrating significant treatment-induced metabolic reprogramming (Fig. 2A-D). To identify specific metabolites responsible for this separation, we employed stringent screening parameters (Variable Importance in Projection VIP > 1, *p* < 0.05), which yielded 16 and 12 significant altered metabolites in positive and negative ion modes, respectively. Hierarchical clustering analysis of these differential metabolites revealed distinct metabolic signatures between the two experimental groups, as visualized in the generated heatmaps (Fig. 2E and F, Table 1 and 2). Subsequent KEGG pathway enrichment analysis of the differential metabolites revealed significant enrichment in pathways associated with fatty acid biosynthesis and lipid metabolism (Fig. 2G and H, Figure S1). This finding suggests that *Piper Longum* primarily exerts its anti-tumor effects by modulating lipid metabolism pathways in GC.

**Table 1:** Identified differentially expressed metabolites between *Piper Longum*-treated and control groups of mice (positive ion)

Metabolite	Rt (s)	m/z	VIP	Fold Change (FC)	*p*-value
14-hydroxy-4z,7z,10z,12e,16z,19z-docosahexaenoic acid	53.8830	327.22971	2.166794	3.1175634	0.0387376
2-piperidinecarboxylic acid, 1-(3,3-dimethyl-1,2-dioxopentyl)-, (1r)-1-(3-aminophenyl)-3-(3,4-dimethoxyphenyl)propyl ester, (2 s)-	116.9660	525.28467	5.093143	0.370605499	0.0449138
8(9)-epoxy-5z,11z,14z,17z-eicosatetraenoic acid	56.1030	301.21458	1.345985	2.893769562	0.02368915
Dibucaine	88.0190	344.22602	2.763175	0.484442226	0.04606615
Fenpropidin	42.3085	274.27336	6.364249	1.330656873	0.02913279
Glu-Thr-Arg	266.8130	203.10140	1.010626	3.043054667	0.04656097
Izenamide c	131.0600	697.35723	3.348568	0.556878253	0.01461173
L-Glutamine	360.4750	188.10273	2.33262	1.911003926	0.02685892
L-Pipecolic acid	303.7740	171.11183	1.334441	1.887532078	0.03270054
L-Pyroglutamic acid	360.5870	171.07562	1.136308	1.78179774	0.0216175
Lpc 16:0	177.0910	496.33958	9.316732	1.329150763	0.04543741
Oleamide	36.6410	282.27955	9.576597	1.425324491	0.03793364
Oleic acid methyl ester	36.2340	247.24478	1.555411	3.02290823	0.0039204
Pipamperone	34.7810	376.25822	1.326257	1.315198688	0.03399774
Pro-thr	382.9450	217.12848	1.252141	1.580272489	0.03276379
Sarcosine	357.3640	131.08109	2.168757	2.621889847	0.01703974

**Table 2:** Identified differentially expressed metabolites between *Piper Longum*-treated and control groups of mice (negative ion)

Metabolite	Rt (s)	m/z	VIP	Fold Change (FC)	*p*-value
12-hydroxy-5z,8z,10e,14z,17z-eicosapentaenoic acid	38.9300	317.21102	1.281008684	1.448886657	0.04909801
12 s-hydroxy-5z,8z,10e,14z-eicosatetraenoic acid	54.0820	319.22758	10.95812383	1.88833113	0.01956616
Cis-4,7,10,13,16,19-docosahexaenoic acid	40.2395	327.23221	5.829916269	1.51578064	0.03505575
Cis-9-palmitoleic acid	41.5150	253.21634	5.075725613	1.79438258	0.02896879
Glu-Met	299.7545	277.07240	1.293583865	0.681260611	0.00125397
Heptadecanoic acid	70.5520	269.24765	1.562608101	2.120945025	7.5187E-05
Madecassoside	165.2975	973.49937	4.425189314	0.643392159	0.04322615
Myristic acid	49.9840	227.20063	2.13799311	1.850439392	0.0405917
N-acetylglutamine	312.2440	187.07126	2.077852559	2.000607754	0.02070789
N-stearoyltaurine	35.5325	390.26585	1.226277853	2.283046955	0.02071366
Palmitic acid	46.7395	255.23282	5.194467319	1.237162208	0.02502684
Prostaglandin b2	110.8045	333.20517	2.598139397	0.538951906	0.02311071

To validate these metabolomic predictions at the transcriptional level, we conducted RT-qPCR analysis of key lipid metabolism regulatory genes in tumor tissues. Consistent with our metabolomic findings, expression levels of SREBP1 and PPARγ significantly downregulated following *Piper Longum* treatment (Fig. 2I). Notably, there was no significant change in expression level of SREBP2 after *Piper Longum* treatment, demonstrating that while this herbal medicine modulates transcription factors associated with fatty acid synthesis and storage, it does not affect cholesterol metabolism-related factors. This finding aligns with our metabolomics data confirming *Piper Longum*'s selective involvement in GC lipid metabolism regulation, particularly in fatty acid synthesis and storage rather than sterol metabolism. These transcriptional suppression corroborates our metabolomic data and provides molecular evidence for *Piper Longum*-induced reprogramming of lipid metabolism in GC. Collectively, our multi-level analysis provides robust evidence that *Piper Longum* inhibits GC growth in vivo primarily through metabolic reprogramming, with particularly emphasis on disruption of lipid metabolism pathways. The observed dose-dependent therapeutic efficacy coupled with minimal systemic toxicity highlights the potential clinical utility of *Piper Longum* as a promising agent for GC treatment.

### Piper Longum alters the gut microbiota in GC mice

**Fig. 3:**
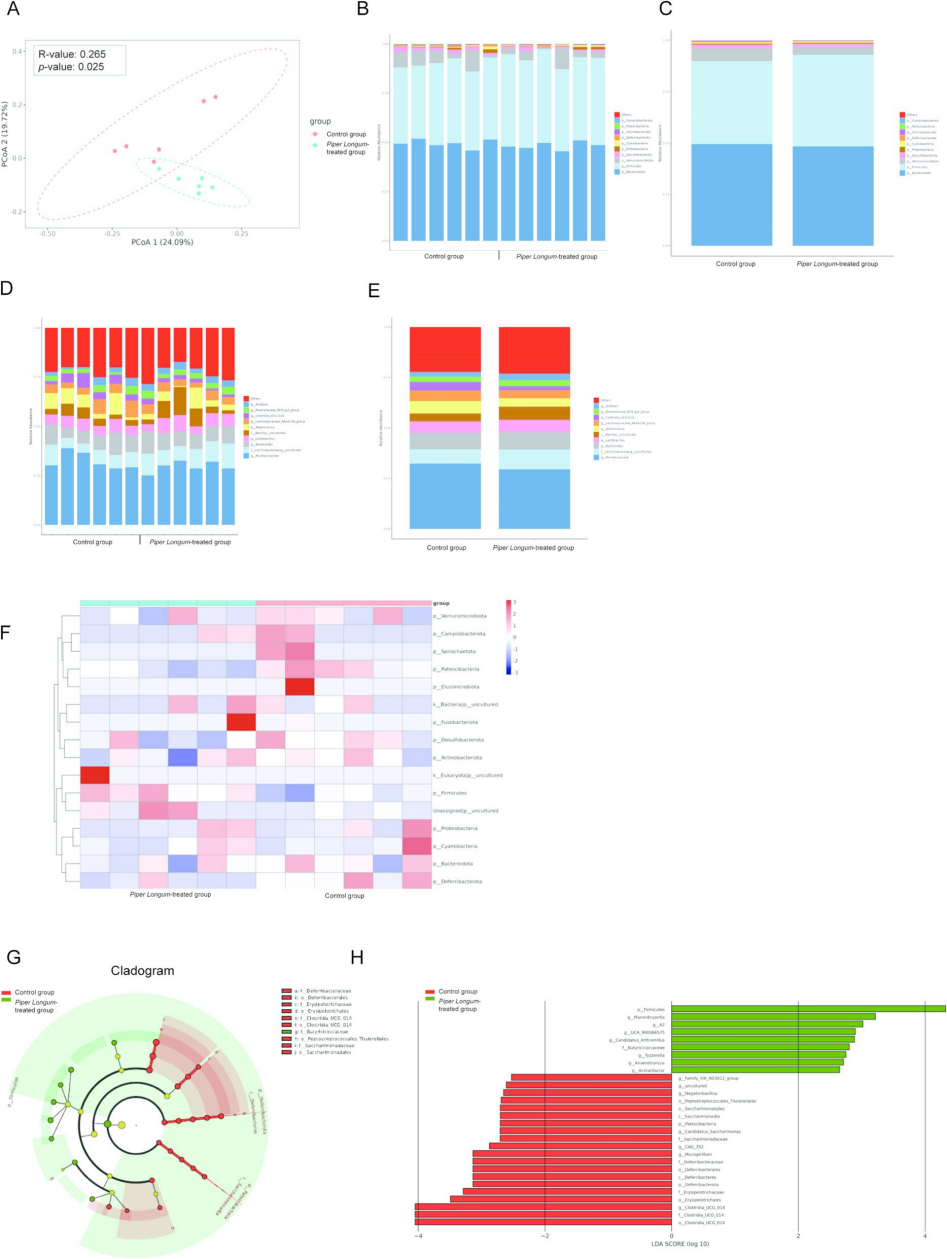
*Piper Longum* Alters the Gut Microbiota in GC Mice. **A**. PCA score plots: C: Red dots represent the control group, D: Blue dots represent the *Piper Longum*-treated group. **B**. The relative abundance bar chart of species at the phylum level for each sample corresponding to each group. **C**. The relative abundance bar chart of species at the phylum level for each group. **D**. Taxonomic changes in gut microbiota at the species level. **E**. Taxonomic changes in gut microbiota at the species level. **F**. Heatmap showing the abundance changes of gut microbiota at the species level. Color intensity corresponds to species abundance, with darker colors indicating higher abundance. **G**. Cladogram (evolutionary branching diagram) illustrating taxonomic levels from phylum to species. Red areas represent the control group, and green areas represent the *Piper Longum*-treated group. Red nodes indicate microbial taxa significantly associated with the control group, while green nodes indicate taxa significantly associated with the *Piper Longum*-treated group. **H**. LDA (Linear Discriminant Analysis) value distribution histogram. Red bars represent the control group, and green bars represent the *Piper Longum*-treated group

To evaluate the impact of *Piper Longum* on intestinal microbial ecology in GC, we performed a systematic analysis of gut microbiota composition and diversity in tumor-bearing mice. Principal coordinate analysis (PCoA) based on weighted UniFrac distances was initially conducted to assess *β*-diversity between treatment groups. The analysis revealed significant separation between the *Piper Longum*-treated and the control groups (Anosim analysis based on Bray–Curtis algorithm: *p*-value: 0.025; R-value: 0.265), indicating substantial restructuring of gut microbial communities following treatment (Fig. 3A). Taxonomic profiling across six hierarchical levels (Phylum, Class, Order, Family, Genus, and Species) revealed treatment-specific signatures in the gut microbiome. At the phylum level, *Piper Longum* administration induced significant shifts in the relative abundance of major bacterial divisions (Fig. 3B). Similar compositional changes were observed at the genus level (Fig. 3C), with several bacterial genera showing differential representation between treatment groups. Species-level analysis further confirmed these compositional alterations, with multiple bacterial species exhibiting significant abundance changes following *Piper Longum* treatment (Fig. 3D and E).

Hierarchical clustering analysis, visualized through heatmaps, demonstrated distinct microbial distribution patterns between control and treatment groups. These alterations were evident starting from the phylum level and extended through all taxonomic ranks (Class, Order, Family, Genus), confirming the comprehensive nature of *Piper Longum*'s impact on gut microbial communities (Fig. 3F). Importantly, despite these substantial compositional changes, analysis of α-diversity indices showed no significant differences between groups (Figure S2A-C). This preservation of overall microbial diversity suggests that *Piper Longum* selectively modulates specific bacterial populations without disrupting the ecological complexity of the gut microbiome, which may contribute to its favorable safety profile. To identify microbial biomarkers specifically associated with *Piper Longum* treatment, we employed Linear discriminant analysis (LEfSe). The resulting cladogram highlighted several differentially abundant microbial taxa (Fig. 3G). Notably, *Piper Longum* treatment was associated with decreased abundance of *Deferribacterales*, *Saccharimonas*, and *Gastranaerophilales* at the higher taxonomic levels (Fig. 3H). These specific microbial signatures may represent potential mechanistic links between gut microbiota modulation and the therapeutic effects of *Piper Longum* (Figure S3).

### Integrated analysis reveals interplay between Piper Longum-induced metabolic reprograming and gut microbiota modulation

**Fig. 4:**
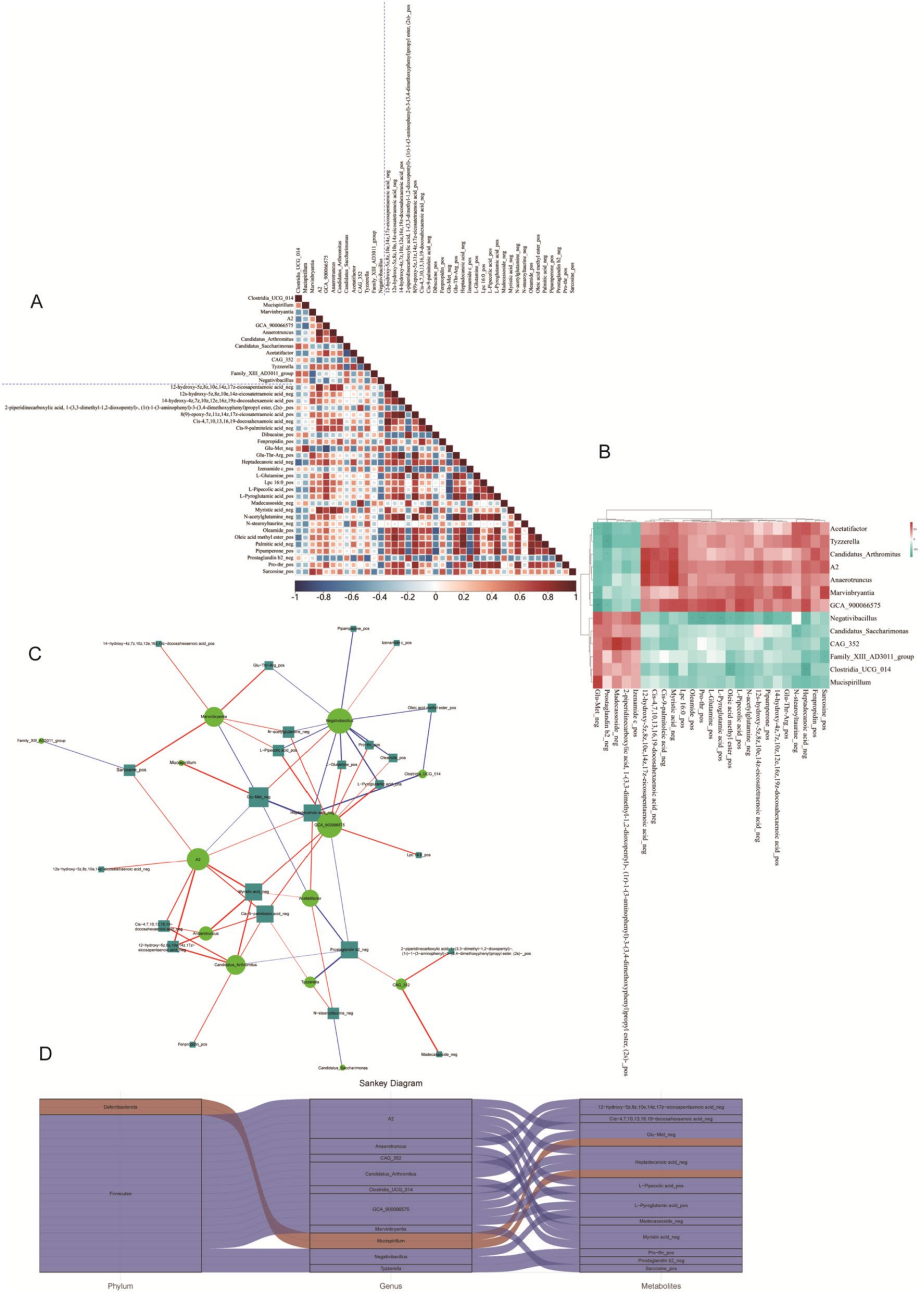
Integrated Analysis of Gut Microbiota and Metabolites. **A**. Spearman correlation coefficient matrix heatmap showing the relationships between significantly differential gut microbiota and significantly differential metabolites. **B**. Hierarchical clustering heatmap of Spearman correlation analysis between significantly differential gut microbiota and significantly differential metabolites. **C**. Network diagram of Spearman correlation analysis between significantly differential gut microbiota and significantly differential metabolites. **D**. Sankey diagram illustrating the relationships between significantly differential gut microbiota and significantly differential metabolites

Having established *Piper Longum*’s distinct effects on both metabolic pathways and gut microbiota composition, we performed comprehensive integrative analyses to elucidate potential mechanistic connections between these complementary biological systems. We constructed correlation coefficient matrices using Spearman analysis to systematically explore associations between differentially abundant microbial taxa and significantly altered metabolites. The resulting heatmap visualization revealed robust correlations between specific microbial signatures and metabolic alterations, suggesting coordinated biological responses to *Piper Longum* treatment rather than independent effects (Fig. 4A). Hierarchical clustering analysis of these differentially abundant microbes and metabolites further substantiated these relationships, demonstrating clear co-clustering patterns that indicate functional associations between microbial communities and metabolic pathways (Fig. 4B). To identify key regulatory nodes within this complex interaction network, we performed Spearman correlation network analysis, focusing on microbial taxa and metabolites with statistically significant (absolute correlation coefficients between 0.5 and 1 and *p*-value < 0.05). The resulting network visualization highlighted critical hub components that may serve as central mediators of *Piper Longum*'s therapeutic activity (Fig. 4C). Further refinement through Sankey diagram analysis revealed specific associations between taxonomic groups and metabolic pathways. Notably, the phylum *Deferribacterota* demonstrated significant correlations with glutamate metabolic pathways and heptadecanoic acid metabolism, providing mechanistic insights into how specific microbial alterations might influence metabolic reprogramming in response to *Piper Longum* treatment (Fig. 4D). Collectively, this integrated multi-omics analysis establishes a compelling framework connecting *Piper Longum*'s dual effects on metabolic pathways and gut microbiota. Rather than representing isolated biological phenomena, our data suggest that these effects are interconnected components of a coordinated therapeutic response.

### Identification of ACOT1 as a key target in Piper Longum-mediated GC treatment through metabolic reprogramming

**Fig. 5:**
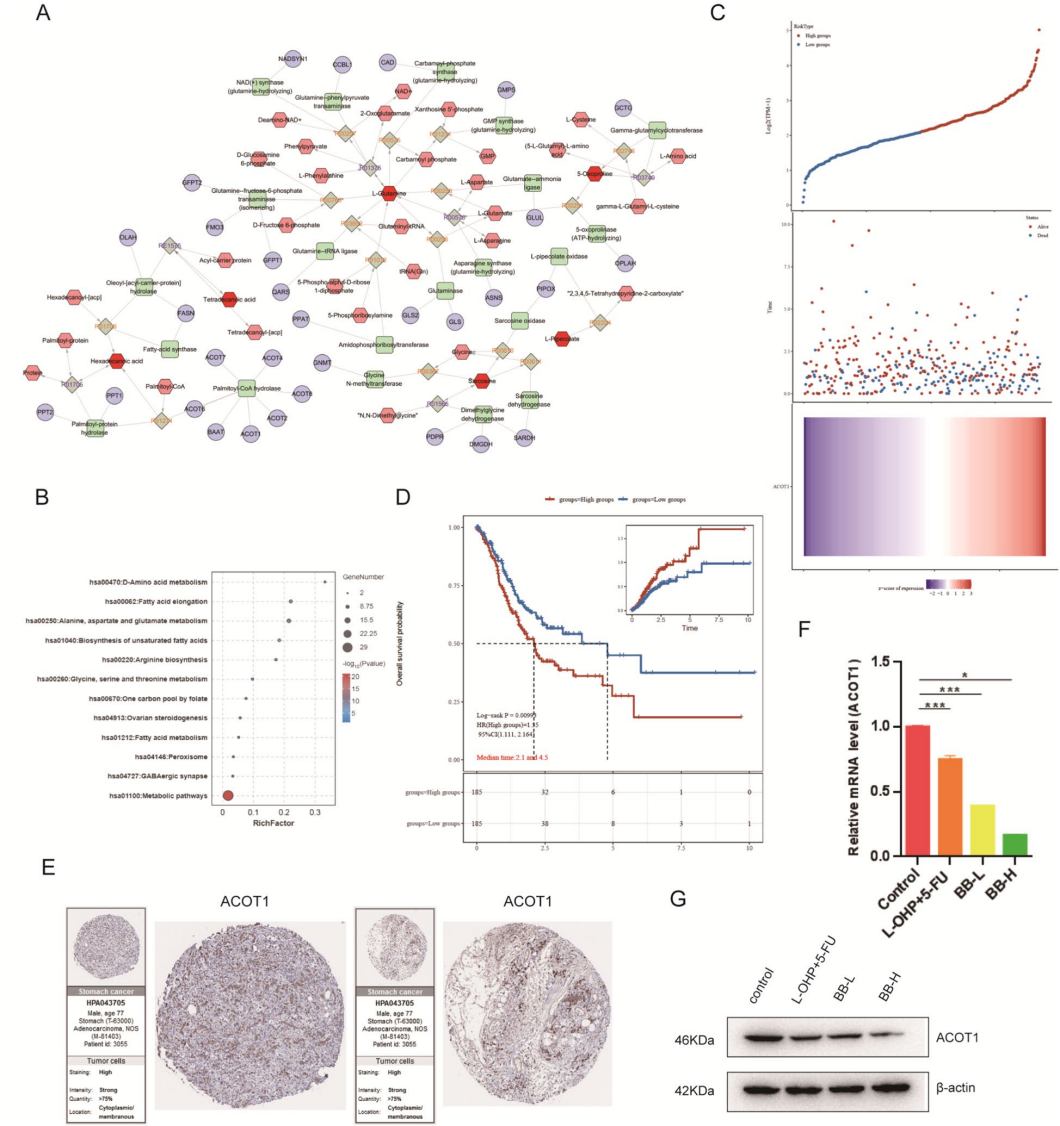
Identification of ACOT1 as a Key Target in *Piper Longum*-Mediated GC Treatment Through Metabolic Reprogramming. **A**. Network interaction analysis of differential metabolites and their associated target proteins. Nodes represent metabolic regulators, and edges represent biochemical reactions. **B**. KEGG enrichment analysis of metabolic regulatory targets. **C**. Correlation analysis between ACOT1 expression and gastric cancer risk coefficients. **D**. Survival prognosis analysis of patients with high ACOT1 expression versus low ACOT1 expression. **E**. Immunohistochemical staining and staging/grading analysis of patients with high ACOT1 expression. **F.** RT-qPCR detection of ACOT1 expression in mouse tumor tissues (*p < 0.05, **p < 0.01). **G**. Western blot analysis of ACOT1 protein expression levels in mouse tumor tissues

To further elucidate key regulatory nodes within the affected metabolic networks, we conducted an in-depth bioinformatic analysis of the metabolomics data obtained from *Piper Longum*-treated mice. Using MetaboAnalyst software, we systematically mapped 28 differential metabolites identified in our untargeted metabolomics analysis to their corresponding KEGG IDs. This process yielded 6 metabolites with definitive KEGG ID annotations for downstream analysis. These annotated metabolites were subsequently integrated into MetScape software to construct a systematic network visualization connecting differential metabolites with their regulatory protein targets (Fig. 5A). KEGG pathway enrichment analysis of this metabolite-target network revealed significant association with D-amino acid metabolism and lipid metabolism (Fig. 5B). This finding consistent with our previous metabolomic results and further substantiated the central role of lipid metabolism reprogramming in *Piper Longum’*s anti-GC machnisms.

To prioritize potential therapeutic targets from this network, we conducted prognostic correlation analysis for each candidate protein using the TCGA database. Among all evaluated targets, only Acyl-CoA Thioesterase 1 (ACOT1) showed a statistically significant correlation between increased expression levels and adverse clinical outcomes in GC patients (Fig. 5C). Kaplan–Meier survival analysis revealed that patients with high ACOT1 expression exhibited poorer overall survival compared to those with low expression, underscoring ACOT1's potential clinical relevance as a prognostic biomarker and therapeutic target in GC (Fig. 5D).

To validate these findings at the tissue level, we examined ACOT1 expression patterns in human GC specimens using immunohistochemical data from the Human Protein Atlas (HPA) database. Consistent with our bioinformatic predictions, elevated ACOT1 expression positively correlated with increased tumor malignancy (Fig. 5E). Finally, to establish a direct link between Piper Longum treatment and ACOT1 expression, we assessed ACOT1 levels in tumor tissues harvested from our in vivo pharmacodynamic experiments described in Fig. 1. Both mRNA and protein levels demonstrated significant downregulation of ACOT1 following *Piper Longum* treatment in a dose-dependent manner (Fig. 5F and G). This finding provides compelling evidence that ACOT1 inhibition represents a critical mechanism through which *Piper Longum* exerts its anti-GC effects via metabolic reprogramming.

### Identification of piperine as the active component of Piper Longum and its role in regulating ACOT1 for GC treatment

**Fig. 6:**
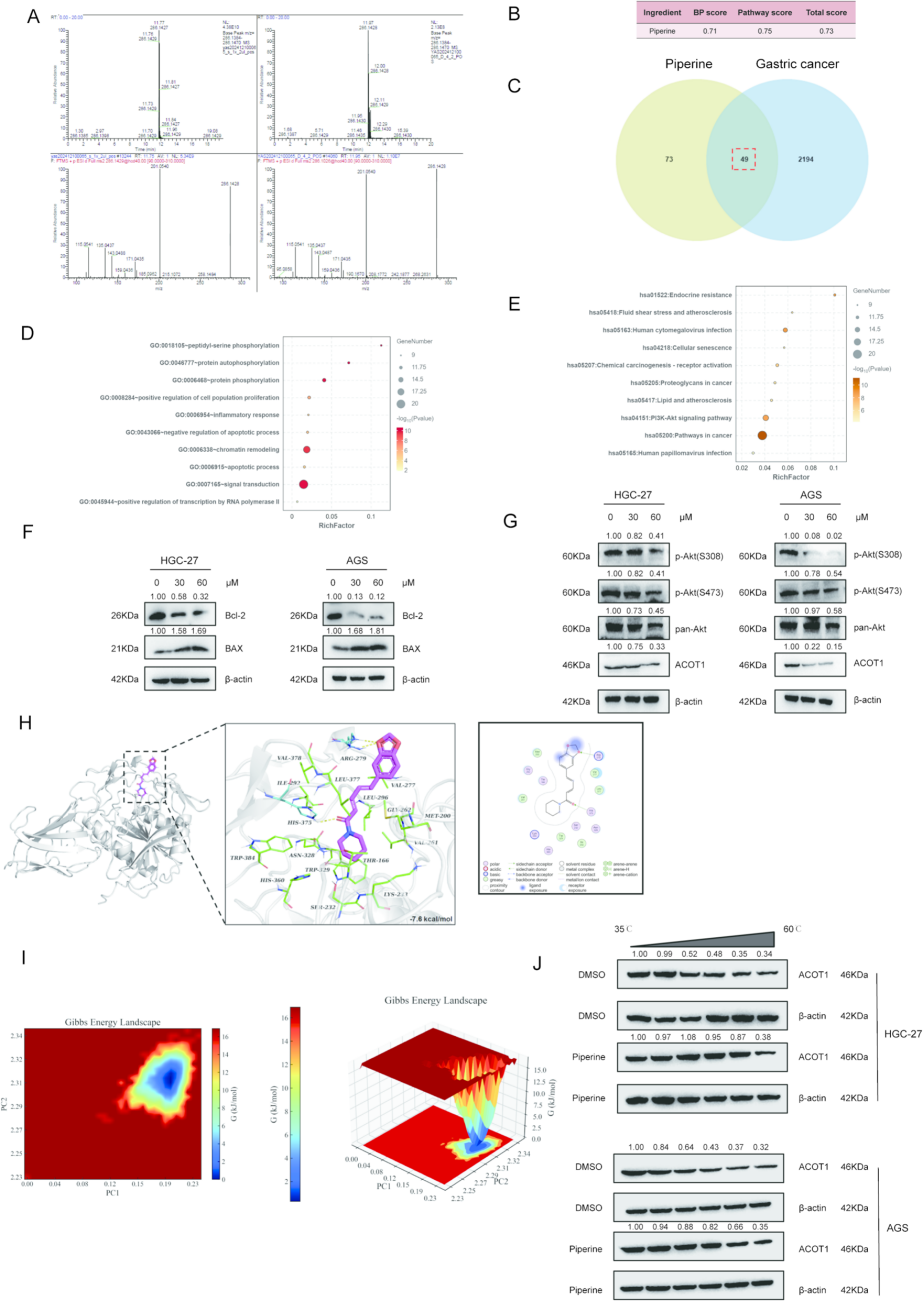
Identification of Piperine as the Active Component of *Piper Longum* and Its Role in Regulating ACOT1 for GC Treatment. **A**. LC–MS results of piperine from *Piper Longum in mice serum samples.* **B**. CHM-FIEFP algorithm analysis between piperine and *Piper Longum*. **C**. Venn diagram showing the intersection of piperine-associated protein targets and significantly differential genes in GC from the TCGA database. **D**. GO enrichment analysis of the 49 overlapping genes. **E**. KEGG enrichment analysis of the 49 overlapping genes. **F** & **G**. Western blot analysis of apoptosis-related proteins (BAX/BCL2), PI3K-Akt pathway proteins, and ACOT1 expression under different piperine treatment concentrations. **H**. Molecular docking results of piperine. **I**. Two- and three-dimensional Gibbs free energy landscapes revealed a stable conformation of the complex, with low Gibbs free energy observed when the RMSD was between 0.19 to 0.22 nm and an Rg value between 2.32 and 2.34 nm (blue regions). **J**. CETSA (Cellular Thermal Shift Assay) to detect the binding interaction between piperine and ACOT1 protein

Liquid chromatography-mass spectrometry (LC–MS) analysis was performed on serum samples from *Piper Longum*-treated and untreated mice to characterize the pharmacologically active components. Through comprehensive metabolite profiling and comparative analysis, piperine was identified as the key bioactive alkaloid in *Piper Longum* with significant presence in the circulation of treated animals (Fig. 6A). Furthermore, we employed a recently developed CHM-FIEFP algorithm [30] (Chinese Herb Medicine-Formula vs. Ingredients Efficacy Fitting & Prediction; http://chm-fiefp.net/) to perform pharmacological fitting analysis specifically comparing the pharmacological efficacy of piperine with the whole *Piper Longum* extract. The results indicated that piperine achieved a high pharmacological fitting score of 0.73 against gastric cancer (Fig. 6B), which represents a strong fitting correlation (where 1.0 indicates perfect fit) indicating that piperine is indeed a key pharmacological ingredient responsible for *Piper Longum*’ anti-gastric cancer effects. To further elucidate the molecular targets of piperine, we employed the TCMSP database, which predicted 122 potential protein targets. We then conducted an integrative multi-database analysis by cross-referencing these targets with gastric cancer-associated genes curated from authoritative databases including GeneCards、MalaCards、OMIM and TTD. Using stringent selection criteria (GeneCards: relevance scores ≥ 2; MalaCards: score threshold ≥ 20), we identified 2243 gastric cancer-related genes. The intersection of these datasets yielded 49 high-confidence target proteins potentially mediating piperine's anti-GC effects (Fig. 6C).

Functional characterization of these 49 targets through Gene Ontology (GO) analysis revealed significant enrichment in biological processes critical to cancer progression, particularly pathways governing cellular proliferation and apoptosis (Fig. 6D). his finding aligns with the observed anti-proliferative effects of *Piper Longum* in our in vivo studies. Complementary KEGG enrichment analysis further demonstrated that these targets were significantly involved in canonical oncogenic signaling networks, with notable enrichment in the PI3K-Akt signaling pathway and lipid metabolism pathways, corroborating our previous metabolomic findings (Fig. 6E).

To validate these computational predictions experimentally, we assessed the effects of piperine on key proteins within the identified pathways. Western blot analysis of apoptosis regulators revealed that piperine treatment decreased anti-apoptotic Bcl-2 expression while simultaneously increasing pro-apoptotic Bax levels (Fig. 6F). Parallel examination of ACOT1 and phosphorylated AKT (at S473 and T308) demonstrated significant reduction in levels with increasing piperine concentrations (Fig. 6G). This suppression of both ACOT1 and activated AKT confirms that piperine modulates GC growth through concurrent inhibition of lipid metabolism and the PI3K-Akt signaling pathways.

The docking analysis revealed that piperine was positioned within the complexes' binding pockets, interacting with various amino acid residues, with binding energies be -7.6 kcal/mol (Fig. 6H). To evaluate stability, Gibbs free energy landscapes were developed in both 2D and 3D. The analyses indicated that the ACOT1-Piperine complex attained a stable conformation with low Gibbs free energy, characterized by an RMSD range of 0.19 to 0.22 nm and an Rg value between 2.32 and 2.34 nm (Fig.  6I and Figure S5).

To determine whether piperine directly interacts with ACOT1 or exerts its effects through intermediate mediators, we performed cellular thermal shift assay (CETSA). The CETSA experimental results showed that piperine treatment enhanced the thermal stability of ACOT1, which was confirmed by the rightward shift of protein bands under increasing temperature conditions (Fig. 6J). Thus, through this integrated approach combining metabolomics, LC–MS, and network pharmacology and experimental validation, we have established piperine as the principal bioactive of *Piper Longum* responsible for its anti-GC effects.

To further clarify the mechanistic role of ACOT1 in piperine's anti-cancer effects, we conducted the comprehensive functional validation experiments using shRNA technology to knock down ACOT1 expression in gastric cancer cells (Figure S6A). Our results demonstrated that ACOT1 knockdown significantly enhanced cellular sensitivity to piperine treatment, as evidenced by increased cytotoxicity and reduced cell viability compared to control shRNA-treated cells (Figure S6B & C). Above findings provide direct functional evidence that ACOT1 plays a critical role in mediating piperine's anti-cancer effects, confirming our proposed mechanistic pathway. Meanwhile, we have also refined our mechanistic understanding through additional RT-qPCR analysis of lipid metabolism-related transcription factors (SREBP1, SREBP2, PPARγ) following both piperine treatment and ACOT1 knockdown experiments. Our key findings demonstrate that SREBP1 and PPARγ expression levels were consistently downregulated under both experimental conditions (Figure S6D). This mechanistic evidence supports a more precise definition of our proposed axis as the "piperine-ACOT1-de novo lipogenesis regulatory pathway."

### Quantum chemical analysis of piperine through density functional theory (DFT) calculation

**Fig. 7:**
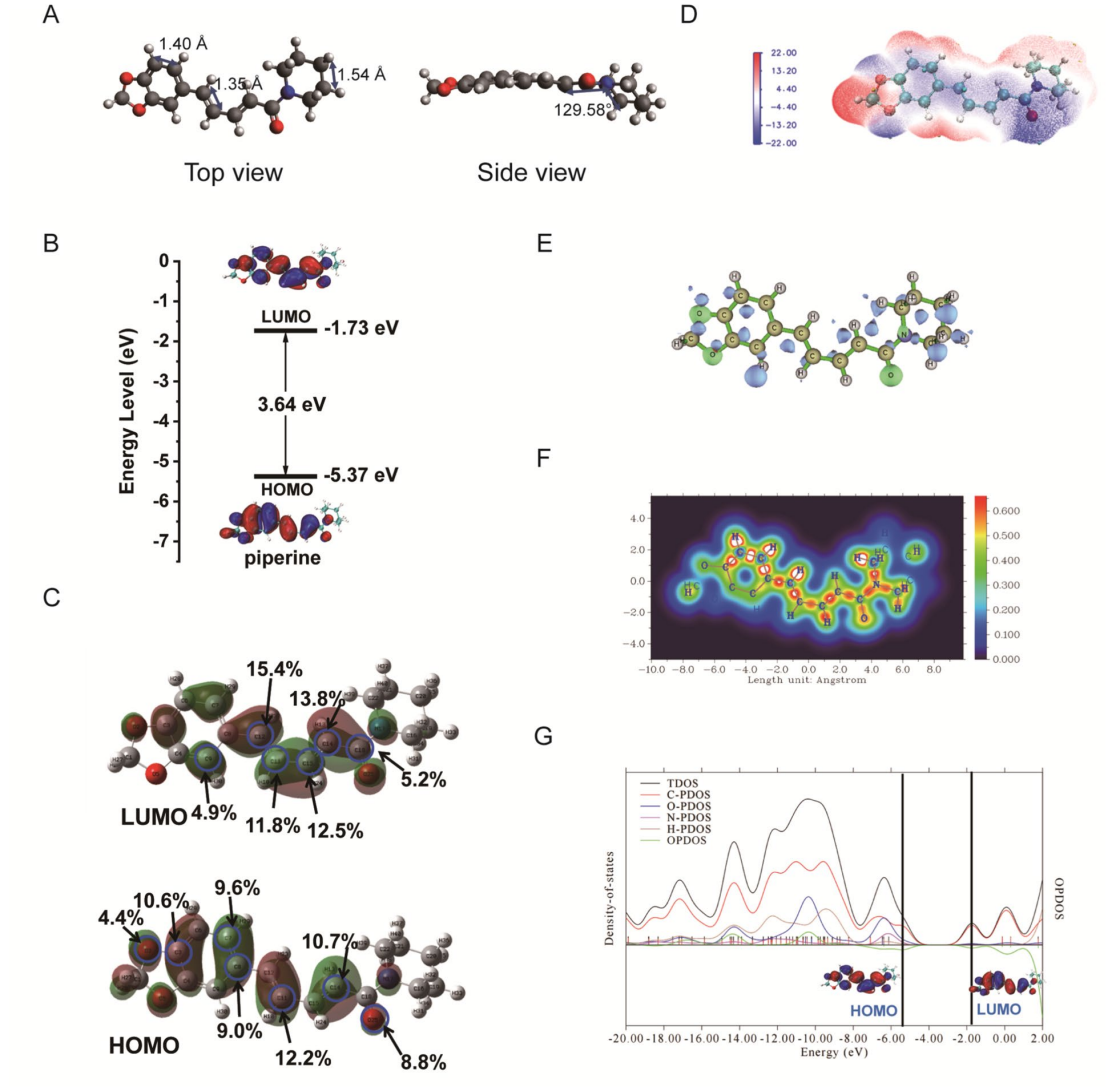
DFT calculations of *piperine*. **A**. The geometrically optimized structures. **B**. The HOMO–LUMO gap. **C**. The orbital composition of HOMO and LUMO. **D**. ESP colored isosurface of ρ = 0.001 a.u. (Cyan and orange spheres correspond to ESP minima and maxima on the surface, respectively. Units are in kcal/mol.). **E**. Fukui Function (f^−^) (The green and blue areas denote positive and negative of f^−^). **F**. LOL-pi and **G**. electron DOS of *piperine*

To elucidate the fundamental structure–activity relationship of piperine at the quantum mechanical level, we performed comprehensive density functional theory (DFT) calculations to characterize its geometric and electronic properties. The optimized molecular structure of *piperine* (Fig. 7A) revealed three distinct structural domains: a methylenedioxyphenyl (MDP) ring, a conjugated double-bond-containing side chain, and a basic piperidine moiety connected via a carbonylamide linkage. Detailed analysis of bond parameters showed characteristic C–C bond lengths of approximately 1.40 Å in the MDP ring, 1.35 Å in the conjugated side chain, and 1.54 Å in the piperidine moiety. The molecule exhibited a non-planar conformation, with atoms of the aromatic and conjugated systems residing nearly in one plane, while the piperidine moiety oriented at a dihedral angle of approximately 129.58° relative to this plane.

Frontier molecular orbitals (FMOs) analysis, which provides critical insights into molecular reactivity and electronic transitions, revealed a HOMO–LUMO energy gap of 3.64 eV for piperine (Fig. 7B). This moderate gap value suggests balanced stability and reactivity, consistent with piperine's biological activity. Visualization of the orbital distributions demonstrated that both HOMO and LUMO were delocalized across the molecule, with the HOMO was predominantly localized on the MDP ring and conjugated double bonds, while LUMO was mainly concentrated on the conjugated double bonds system. Quantitative orbital composition analysis was employed to precisely identify potential electrophilic (nucleophilic) sites (Fig. 7C). Atoms C3, C7, C8, C11, C14, O2, and O25 exhibited significant contributions to the HOMO, indicating their propensity as electrophilic reaction sites. On the other hand, atoms C9, C11, C12, C14, C15, and C18 contributed more significantly to the LUMO, suggesting their potential role as nucleophilic sites. This atomic-level reactivity mapping provides valuable insights for understanding piperine's interactions with biological targets like ACOT1.

Molecular electrostatic potential (ESP) mapping, which visualizes charge distribution across the molecular surface [31, 32], revealed distinct electronegative and electropositive regions. As shown in Fig. 7D, the negative ESP in blue was related to electrophilic reactivity and positive regions in red were related to nucleophilic reactivity. The amide carbonyl oxygen (O25) exhibited the most pronounced electronegative potential (blue region), representing the preferential site for electrophilic attack, followed by the oxygen atoms in the MDP ring. This finding was further corroborated by Donor Fukui Function (f-) analysis (Fig. 7E), which demonstrated that oxygen atoms in piperine exhibited superior charge-donating capability (green regions), particularly the amide carbonyl oxygen.

To evaluate π-electrons delocalization, which significantly influences molecular stability and reactivity, we generated electron localization function (ELF) maps at an isovalue of 0.55 (Fig. 7F). The visualization clearly illustrated the delocalization pathways of π-electrons above and below the molecular plane [33, 34]. Notably, electron delocalization occurred more efficiently along shorter C–C bonds (indicated by broader isosurfaces), enhancing reactivity at these positions. In contrast, delocalization across longer C–C bonds is relatively hindered (narrower isosurfaces or complete breaks), limiting reactivity at these sites.

Finally, comprehensive electronic structure analysis through density of states (DOS) calculations provided detailed insights into the orbital contributions from individual atoms (Fig. 7G). The total DOS (TDOS), partial DOS (PDOS), and overlap population DOS (OPDOS) revealed that the HOMO energy level primarily originated from hybrid contributions of carbon and oxygen atomic orbitals. Conversely, the LUMO was predominantly composed of carbon orbitals, with minimal contributions from other atoms. The OPDOS (green line) indicated that the HOMO exhibited C-O antibonding character (negative OPDOS values), while strong bonding interactions existed between carbon, oxygen, and nitrogen atoms below the HOMO energy level (positive OPDOS values). Interestingly, the LUMO displayed nonbonding character between these atoms, as evidenced by near-zero OPDOS values at the LUMO energy level, suggesting that LUMO occupancy would not significantly alter the bonding characteristics of *piperine*.

These comprehensive quantum chemical analyses provide unprecedented molecular-level insights into piperine’s electronic structure and reactivity patterns, establishing a foundation for understanding its interactions with biological targets and informing structure-based drug design approaches for optimizing piperine derivatives as potential gastric cancer therapeutics.

## Discussion

Our comprehensive investigation into the anti-gastric cancer properties of *Piper Longum* has revealed several significant and interconnected mechanisms that contribute to its therapeutic efficacy. These findings not only expand our understanding of traditional medicine's molecular basis but also identify promising targets for gastric cancer treatment.

The in vivo efficacy of *Piper Longum* against gastric cancer demonstrated in our xenograft model provides compelling evidence for its therapeutic potential. Notably, high-dose *Piper Longum* treatment demonstrated comparable or even superior tumor suppression relative to conventional first-line chemotherapy (L-OHP + 5-FU), while exhibiting minimal systemic toxicity. This favorable efficacy-safety profile represents a significant advantage over current treatment options, which are often limited by severe adverse effects [35, 36]. Our findings align with previous studies reporting anti-proliferative effects of various Piper species in different cancer models [37, 38], but extend these observations specifically to gastric cancer with robust in vivo validation.

The most significant contribution of our study lies in the elucidation of the mechanistic basis for *Piper Longum*'s anti-cancer effects through comprehensive multi-omics integration. Our metabolomic analysis revealed that *Piper Longum* treatment significantly reprograms metabolic pathways in gastric cancer, with particular emphasis on lipid metabolism. This finding is particularly noteworthy given the emerging recognition of altered lipid metabolism as a hallmark of cancer progression [39, 40]. Specifically, the downregulation of key lipid metabolism regulators SREBP1 and PPARγ following *Piper Longum* treatment suggests disruption of lipogenic programs that sustain cancer cell proliferation. This metabolic intervention represents a novel therapeutic approach that targets the distinct metabolic vulnerabilities of cancer cells.

Concurrent with metabolic reprogramming, we observed significant alterations in gut microbiota composition following *Piper Longum* treatment. The preservation of α-diversity coupled with distinct shifts in community structure suggests that *Piper Longum* selectively modulates specific bacterial populations rather than causing broad dysbiosis. Particularly noteworthy was the decreased abundance of Deferribacterales, which has been previously associated with gastrointestinal inflammation and cancer progression [41–43]. Our integrated analysis establishing correlations between specific microbial taxa and metabolic alterations provides strong evidence for a microbiota-metabolism axis in mediating *Piper Longum*'s therapeutic effects. This finding contributes to the growing appreciation of the microbiome's role in cancer development and treatment response.

The identification of ACOT1 as a key molecular target represents a critical advancement in understanding the specific mechanisms underlying *Piper Longum*'s efficacy. ACOT1, an acyl-CoA thioesterase that regulates fatty acid metabolism by hydrolyzing acyl-CoAs to free fatty acids and CoA [44], emerged as a significant prognostic factor in gastric cancer from our TCGA analysis. The dose-dependent downregulation of ACOT1 following *Piper Longum* treatment mechanistically links the observed metabolic alterations with therapeutic outcomes. This finding is consistent with recent studies implicating ACOT1 in cancer metabolism and progression, but notably establishes its specific relevance in gastric cancer, where it has been previously underexplored.

Our identification of piperine as the principal bioactive component responsible for ACOT1 inhibition and anti-cancer effects provides a critical link between traditional phytotherapeutic approaches and modern targeted therapies. CETSA results indicate that piperine engages ACOT1 through a specific molecular mechanism rather than nonspecific cytotoxicity; nevertheless, additional biophysical assays (e.g., SPR or ITC) are required to confirm direct binding. Furthermore, the observed modulation of the PI3K-Akt signaling pathway and apoptotic regulators (Bcl-2/Bax) by piperine establishes a comprehensive mechanism connecting lipid metabolism disruption with cell death pathways. These findings align with previous studies reporting diverse anti-cancer effects of piperine [21, 22], but uniquely establish ACOT1 as a specific molecular target.

The quantum chemical analysis of piperine through DFT calculations provides unprecedented insights into its structure–activity relationship at the atomic level. The moderate HOMO–LUMO gap of 3.64 eV explains piperine's balanced stability and reactivity, while the identification of specific electrophilic and nucleophilic sites offers valuable information for understanding its interaction with ACOT1. Particularly noteworthy is the high electronegative potential at the amide carbonyl oxygen (O25), which likely plays a crucial role in molecular recognition and binding. These detailed structural insights establish a foundation for rational drug design approaches aimed at developing optimized piperine derivatives with enhanced specificity and potency against ACOT1.

Our findings have several important translational implications. First, they provide mechanistic validation for the traditional use of *Piper Longum* in cancer treatment, establishing a scientific basis for ethnopharmacological approaches. Second, the identification of ACOT1 as a novel therapeutic target in gastric cancer expands the repertoire of potential interventions beyond conventional cytotoxic approaches. Third, the elucidation of piperine's structure–activity relationship offers opportunities for medicinal chemistry optimization to enhance its pharmacological properties.

Despite these significant advances, several limitations and future directions warrant consideration. While our study demonstrates *Piper Longum*'s efficacy in mouse models, clinical validation in human patients remains necessary. Additionally, comprehensive pharmacokinetic analysis of piperine and potential metabolites would further inform optimal dosing strategies. Future studies should also explore potential synergistic interactions between piperine and conventional chemotherapeutics, as combination approaches [45] might enhance efficacy while reducing toxicity.

## Conclusions

**Fig. 8:**
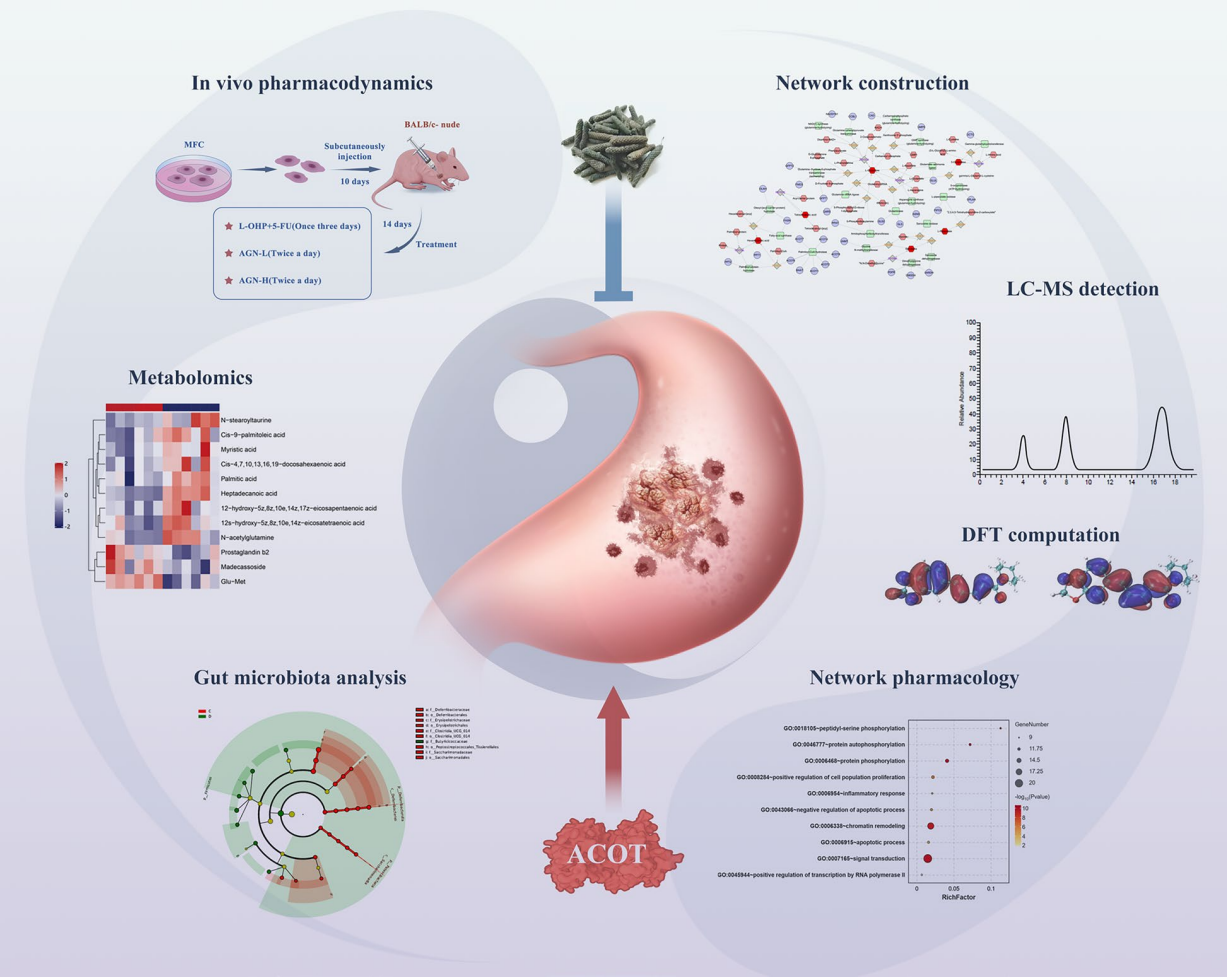
The schematic diagram of *Piper Longum* 's mechanism in treating GC

In conclusion, our comprehensive investigation establishes that *Piper Longum* exerts potent anti-gastric cancer effects through a multi-modal mechanism involving metabolic reprogramming and gut microbiota modulation, with ACOT1 serving as a central molecular target. Piperine emerges as the principal bioactive component, directly interacting with ACOT1 to disrupt lipid metabolism and activate apoptotic pathways (Fig. 8). These findings not only provide mechanistic insights into traditional medicine but also identify promising therapeutic targets and lead compounds for GC treatment, potentially addressing the critical need for more effective and less toxic interventions for this devastating malignancy.

## Supplementary Information

Additional file 1: Figure S1 Pathway enrichment analysis of differential metabolites

Additional file 2: Figure S2 (A-C) α-Diversity analysis of gut microbiota under three different conditions.

Additional file 3: Figure S3 Pathway enrichment analysis of differential gut microbiota grouping.

Additional file 4: Figure S4 Changes in ACOT1 mRNA expression levels in GC after treatment with different concentrations of *piperine* (**p < 0.01, ***p < 0.001).

Additional file 5: Figure S5 Molecular dynamics simulation analysis. A. RMSD fluctuations demonstrated the stabilization of the complex, with distinct curves for ACOT1 (black), piperine (red), and the ACOT1-piperine complex (blue). B. RMSF analysis highlighted significant fluctuations. C. Fluctuations in the number of hydrogen bonds (ranging from 1 to 7) during the simulation highlighted conformational changes and variations in ligand binding.

Additional file 6: Figure S6 Biological functional experimental verification. A. Western blot analysis was performed to validate the knockdown efficiency of ACOT1 at the protein level. B. CCK-8 assays were conducted to evaluate cytotoxicity under three experimental conditions: (1) piperine treatment alone, (2) ACOT1 knockdown alone, and (3) combined piperine treatment with ACOT1 knockdown. The results demonstrated significantly enhanced drug sensitivity in the ACOT1 knockdown group treated with piperine (**p<0.01, ***p<0.001). C. The colony formation assay was performed to assess cell viability under three experimental conditions: (1) piperine treatment alone, (2) ACOT1 knockdown alone, and (3) combined piperine treatment with ACOT1 knockdown, revealing enhanced drug sensitivity to piperine in the ACOT1 knockdown group (**p<0.01, ***p<0.001). D. The RT-qPCR assay to examine the expression changes of lipogenesis-related genes SREBP1 and PPARγ under two experimental conditions: (1) after treatment with different concentrations of piperine, and (2) following ACOT1 knockdown, with both showing statistically significant alterations (**p<0.01).

Additional file 7: Table S1 Antibodies. Table S2 RT-qPCR primer.

## Data Availability

No datasets were generated or analysed during the current study.

## Abbreviations

GC: Gastric cancer
TCM: Traditional Chinese medicine
BB: *Piper Longum*
PPI: Protein–protein interaction
FBS: Foetal bovine serum
OPLS-DA: Orthogonal partial least squares discriminant analysis
VIP: Variable importance in the projection
DFT: Density functional theory
