# Supplementary figures and images for: Multi-omics analysis reveals ACOT1 as the key target of piperine in Piper Longum-mediated gastric cancer treatment
Source: Chin Med. 2025 Aug 25;20:133. doi: 10.1186/s13020-025-01186-y (PMC12376458; doi:10.1186/s13020-025-01186-y)

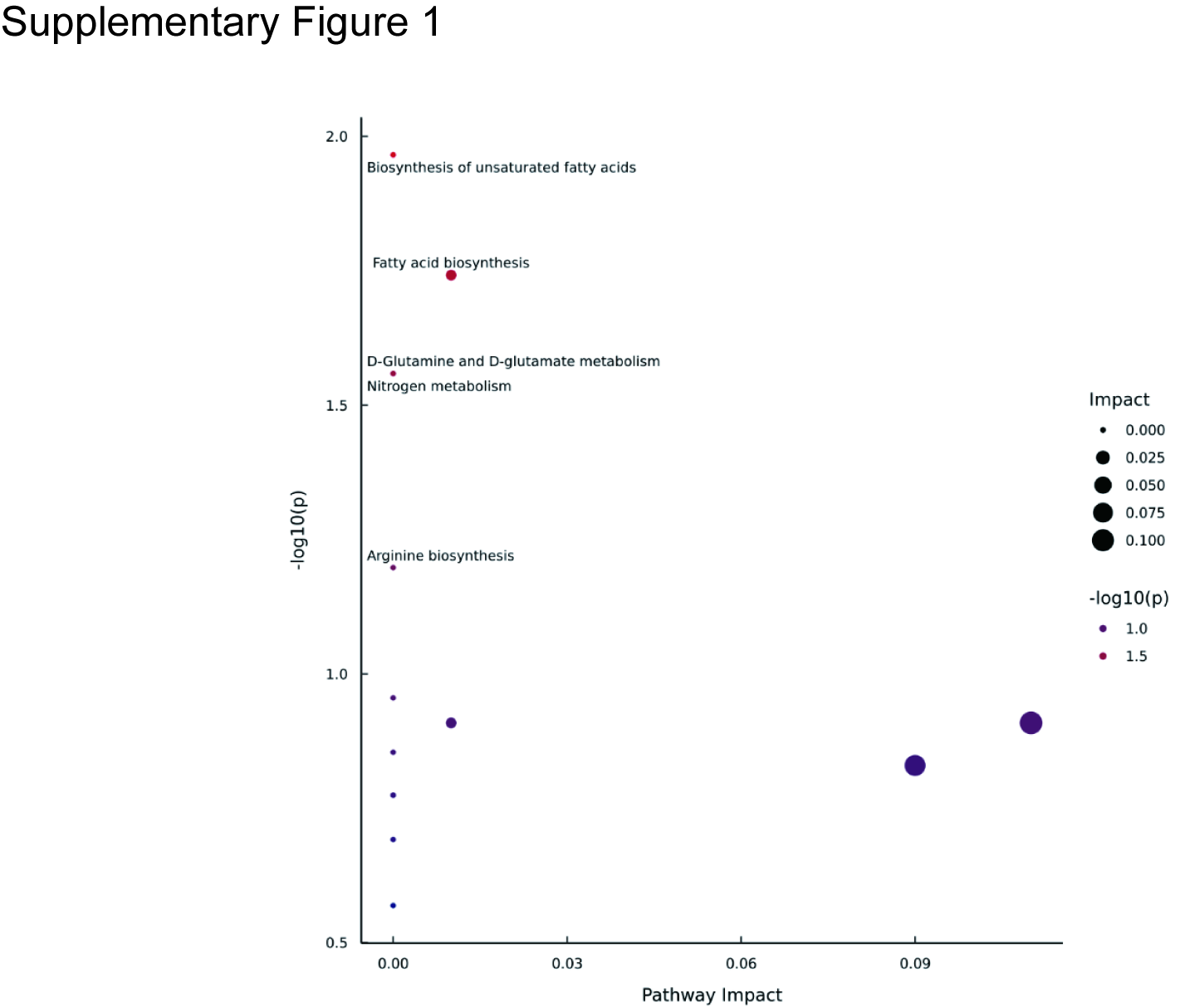

Supplement: Supplementary file 1 — Additional file 1: Figure S1 Pathway enrichment analysis of differential metabolites [file 13020_2025_1186_MOESM1_ESM.tif]

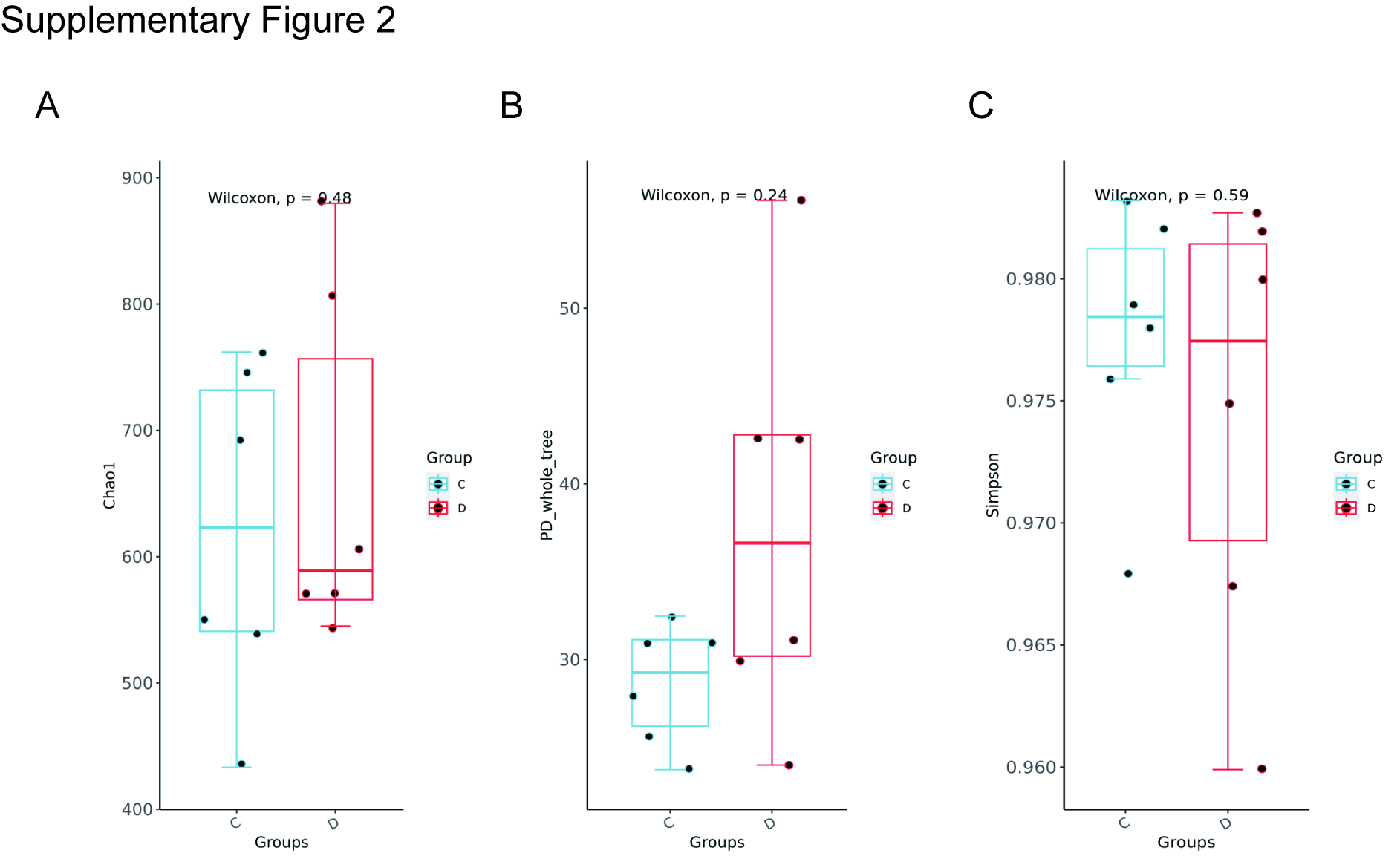

Supplement: Supplementary file 2 — Additional file 2: Figure S2 (A-C) α-Diversity analysis of gut microbiota under three different conditions. [file 13020_2025_1186_MOESM2_ESM.tif]

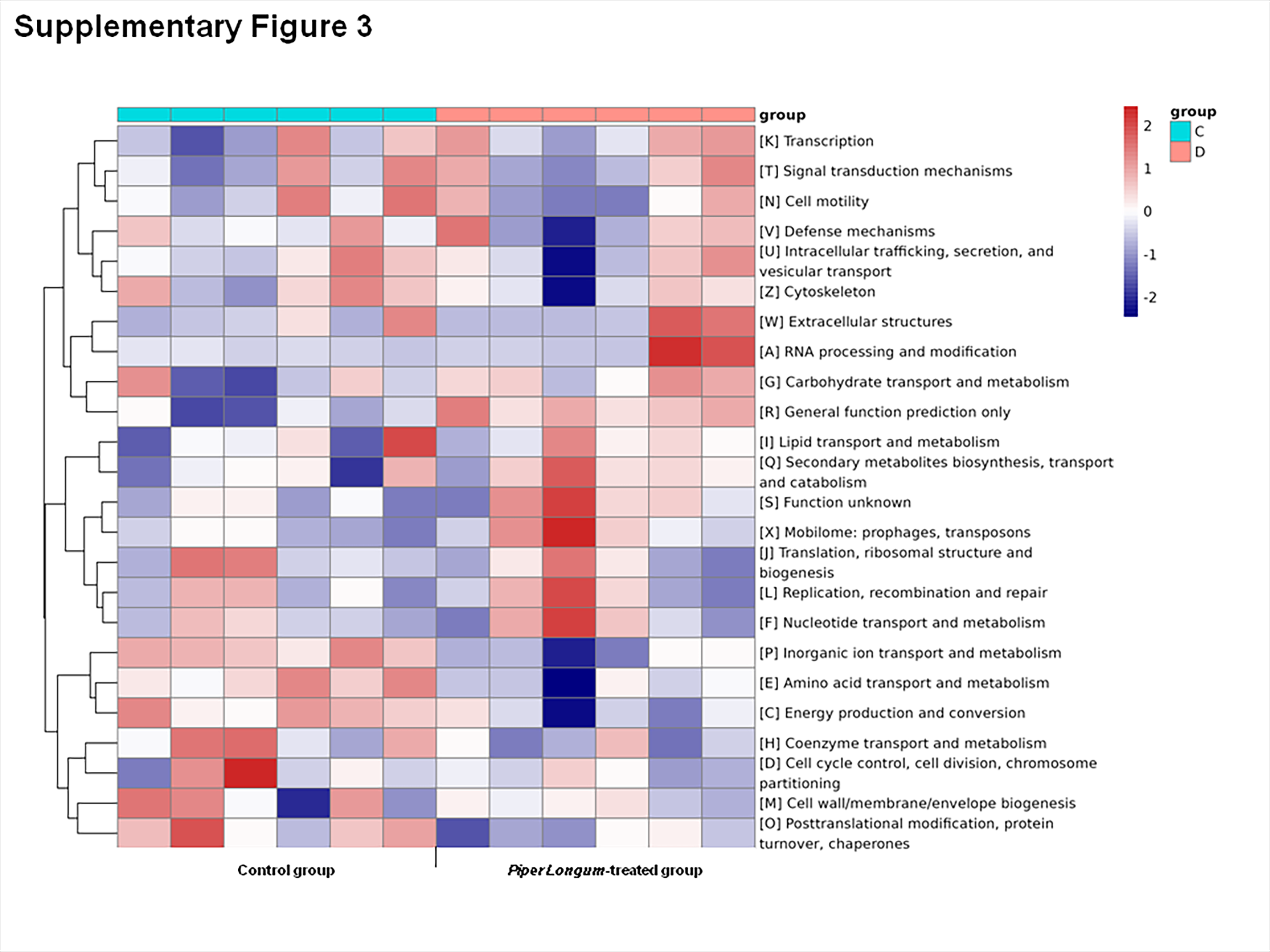

Supplement: Supplementary file 3 — Additional file 3: Figure S3 Pathway enrichment analysis of differential gut microbiota grouping. [file 13020_2025_1186_MOESM3_ESM.tif]

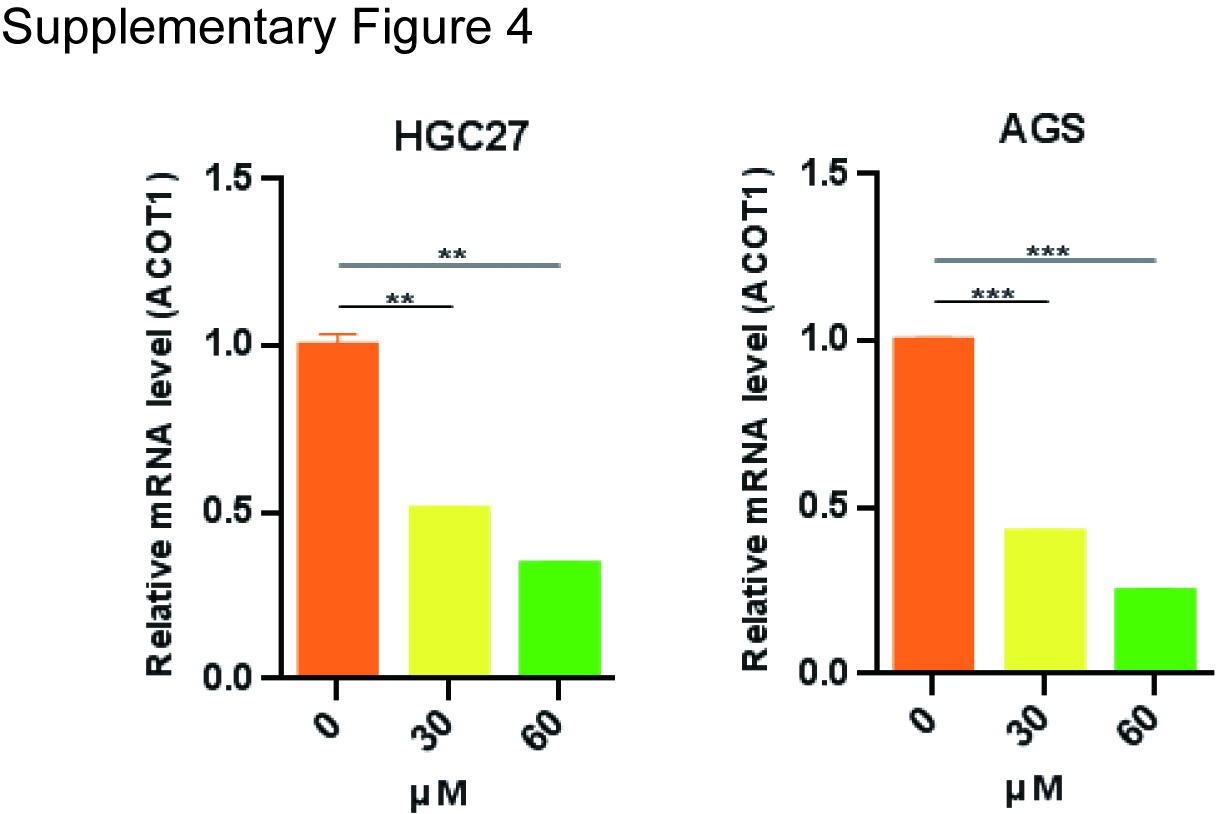

Supplement: Supplementary file 4 — Additional file 4: Figure S4 Changes in ACOT1 mRNA expression levels in GC after treatment with different concentrations of piperine (**p < 0.01, ***p < 0.001). [file 13020_2025_1186_MOESM4_ESM.tif]

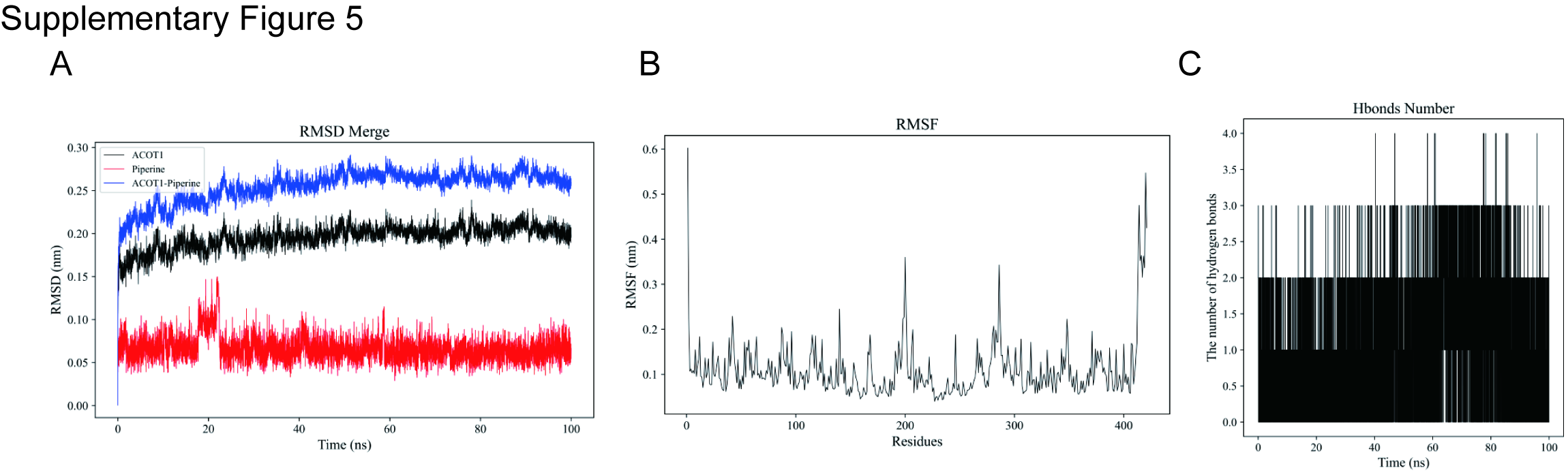

Supplement: Supplementary file 5 — Additional file 5: Figure S5 Molecular dynamics simulation analysis. A. RMSD fluctuations demonstrated the stabilization of the complex, with distinct curves for ACOT1 (black), piperine (red), and the ACOT1-piperine complex (blue). B. RMSF analysis highlighted significant fluctuations. C. Fluctuations in the number of hydrogen bonds (ranging from 1 to 7) during the simulation highlighted conformational changes and variations in ligand binding. [file 13020_2025_1186_MOESM5_ESM.tif]

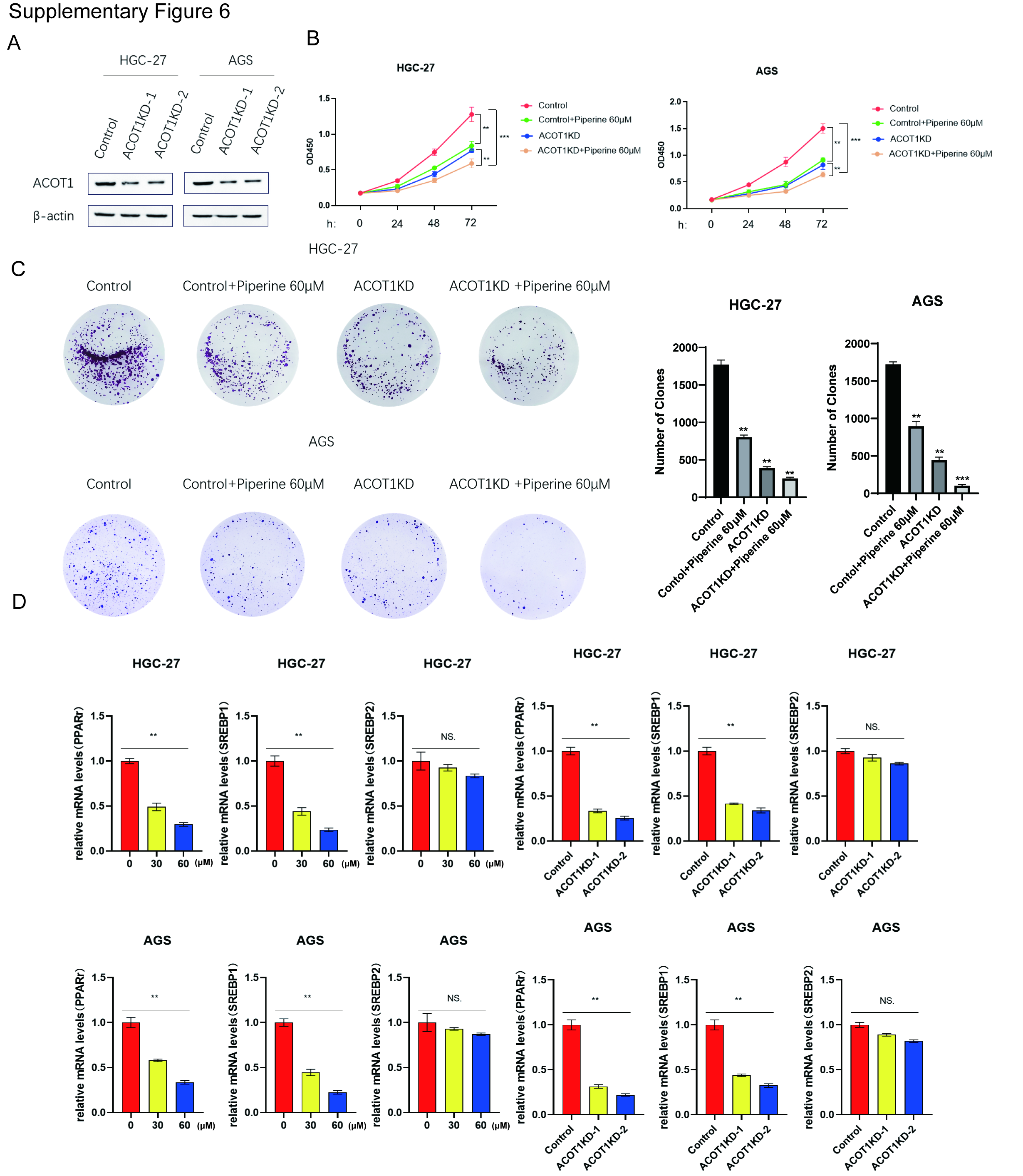

Supplement: Supplementary file 6 — Additional file 6: Figure S6 Biological functional experimental verification. A. Western blot analysis was performed to validate the knockdown efficiency of ACOT1 at the protein level. B. CCK-8 assays were conducted to evaluate cytotoxicity under three experimental conditions: (1) piperine treatment alone, (2) ACOT1 knockdown alone, and (3) combined piperine treatment with ACOT1 knockdown. The results demonstrated significantly enhanced drug sensitivity in the ACOT1 knockdown group treated with piperine (**p<0.01, ***p<0.001). C. The colony formation assay was performed to assess cell viability under three experimental conditions: (1) piperine treatment alone, (2) ACOT1 knockdown alone, and (3) combined piperine treatment with ACOT1 knockdown, revealing enhanced drug sensitivity to piperine in the ACOT1 knockdown group (**p<0.01, ***p<0.001). D. The RT-qPCR assay to examine the expression changes of lipogenesis-related genes SREBP1 and PPARγ under two experimental conditions: (1) after treatment with different concentrations of piperine, and (2) following ACOT1 knockdown, with both showing statistically significant alterations (**p<0.01). [file 13020_2025_1186_MOESM6_ESM.tif]
