# Supplementary material for: Multi-omics analysis reveals ACOT1 as the key target of piperine in Piper Longum-mediated gastric cancer treatment
Source: Chin Med. 2025 Aug 25;20:133. doi: 10.1186/s13020-025-01186-y (PMC12376458; doi:10.1186/s13020-025-01186-y)
Supplement: Supplementary file 7 — Additional file 7: Table S1 Antibodies. Table S2 RT-qPCR primer. [file 13020_2025_1186_MOESM7_ESM.doc]

Table S1 Antibodies

| Proteintech Group (China) | Bcl-2 (12789-1-AP, 100 μL, 1:1000) |
| --- | --- |
| Proteintech Group (China) | Bax (50599-2-Ig, 100 μL, 1:1000) |
| Proteintech Group (China) | phosphorylated-Akt (T308) (29163-1-AP, 100 μL, 1:1000) |
| Proteintech Group (China) | phosphorylated-Akt (S473) (66444-1-Ig, 100 μL, 1:1000) |
| Proteintech Group (China) | β-actin (66009-1-Ig, 100 μL, 1:1000) |
| Cell Signaling Technology (USA) | pan-Akt (9272S, 100 μL, 1:1000) |
| Abcam (UK) | ab100915 (100μg, 1:500) |

Table S2 RT-qPCR primer

|  | Forward primer | Reverse primer |
| --- | --- | --- |
| Rp0 | TTCATTGTGGGAGCAGAC | CAGCAGTTTCTCCAGAGC |
| SREBP1 | GCGCCTTGACAGGTGAAGTC | GCCAGGGAAGTCACTGTCTTG |
| SREBP2 | AGAATGTCCTTCTGATGTCC | GGAGAGTCTGGCTCATCTT |
| PPAR r | AGACCACTCCCACTCCTTTG | ATGAGGGAGTTGGAAGGCTC |
| ACOT1 | AGTCACATTCGGGATGAGCT | CTCAACTGTCTCTGCTTGTT |
